# Decoding the Semantic Content of Natural Movies from Human Brain Activity

**DOI:** 10.3389/fnsys.2016.00081

**Published:** 2016-10-07

**Authors:** Alexander G. Huth, Tyler Lee, Shinji Nishimoto, Natalia Y. Bilenko, An T. Vu, Jack L. Gallant

**Affiliations:** ^1^Helen Wills Neuroscience Institute, University of CaliforniaBerkeley, Berkeley, CA, USA; ^2^Bioengineering Graduate Group, University of CaliforniaBerkeley, Berkeley, CA, USA; ^3^Department of Psychology, University of CaliforniaBerkeley, Berkeley, CA, USA

**Keywords:** fMRI, decoding, natural images, WordNet, structured output

## Abstract

One crucial test for any quantitative model of the brain is to show that the model can be used to accurately decode information from evoked brain activity. Several recent neuroimaging studies have decoded the structure or semantic content of static visual images from human brain activity. Here we present a decoding algorithm that makes it possible to decode detailed information about the object and action categories present in natural movies from human brain activity signals measured by functional MRI. Decoding is accomplished using a hierarchical logistic regression (HLR) model that is based on labels that were manually assigned from the WordNet semantic taxonomy. This model makes it possible to simultaneously decode information about both specific and general categories, while respecting the relationships between them. Our results show that we can decode the presence of many object and action categories from averaged blood-oxygen level-dependent (BOLD) responses with a high degree of accuracy (area under the ROC curve > 0.9). Furthermore, we used this framework to test whether semantic relationships defined in the WordNet taxonomy are represented the same way in the human brain. This analysis showed that hierarchical relationships between general categories and atypical examples, such as *organism* and *plant*, did not seem to be reflected in representations measured by BOLD fMRI.

## Introduction

In the past decade considerable interest has developed in decoding stimuli or mental states from brain activity measured using functional magnetic resonance imaging (fMRI). Early results in this field (Kay et al., 2008; Mitchell et al., 2008; Naselaris et al., 2009; Nishimoto et al., 2011) have created substantial excitement over the prospect of futuristic non-invasive brain-computer interfaces that could perform “brain reading.” These studies have shown that substantially more information can be recovered from BOLD fMRI than many had previously believed (Kay et al., 2008). Furthermore, one recent study from our laboratory showed that it is possible to decode the appearance of rapidly changing natural movies using fMRI (Nishimoto et al., 2011), challenging the received wisdom that fMRI is only suitable for studying slow phenomena. Here we extend our previous work by decoding which categories of objects and actions are present in natural movies.

Brain decoding can be viewed as the problem of finding the stimulus, *S*, that is most likely to have evoked the observed BOLD responses, *R*, under the probability distribution *P(S | R)*. To date, two general approaches have been been used to solve this problem: Bayesian decoding and direct decoding. In Bayesian decoding one constructs an explicit model of *P(R | S)* in order to predict the response based on the stimulus. Then, Bayes' rule is used to invert the conditional probability: *P(S | R)* = *P(R | S) P(S) / P(R)*. This approach has been used to decode the visual appearance and semantic category of static natural images (Naselaris et al., 2009), the visual appearance of natural movies (Nishimoto et al., 2011), and the semantic category of isolated visual objects or words (Mitchell et al., 2008). However, Bayesian decoding requires the construction of a prior distribution over stimuli, *P(S)*, and this is impractical when the decoding space is large (e.g., when decoding natural scenes or movies). In some cases, this issue can be solved by using a large empirical prior (Naselaris et al., 2009; Nishimoto et al., 2011). However, we have no way to estimate the empirical prior for categories appearing in natural movies. This makes it difficult to apply the Bayesian decoding framework to this problem.

The other popular approach to this problem is direct decoding. In this approach, one constructs an explicit model of *P(S | R)* that directly predicts the stimulus based on the response. Direct decoding has been used to decode which of two visual categories is being viewed (Haxby et al., 2001; Carlson et al., 2003; Cox and Savoy, 2003), which of two categories a subject is dreaming about (Horikawa et al., 2013), and which objects are present in static natural visual scenes (Stansbury et al., 2013). However, for several reasons direct decoding is usually not optimal for decoding objects and actions in natural scenes from brain activity. First, direct decoding implicitly assumes that each decoded feature is independent, but objects and actions in natural scenes tend to be correlated with one another (although recent work from our lab has shown that it is possible to work around this issue by transforming the stimuli into a feature space where the independence assumption is valid Stansbury et al., 2013). Second, each object or action has many potential category labels that are related in a nested, hierarchical structure. For example, a *1993 Mercury Sable* could also be called a *station wagon*, a *car*, a *motor vehicle*, etc. These labels are not independent and so should not be decoded independently. One solution to this issue would be to decode only one label in the hierarchy, such as the basic-level category (Rosch et al., 1976), which in this example would likely be *car*. However, a basic level category decoder would ignore fMRI signals related to subordinate categories (such as *station wagon* or *1993 Mercury Sable*), which might carry additional information about the visual scene. Furthermore, obtaining basic-level category labels would require extensive manual labeling from multiple observers. For these reasons, here we elected to use a different approach in which we decoded categories at many different levels within a hierarchy simultaneously.

Our direct decoding approach, hierarchical logistic regression (HLR), decodes which object and action categories are present in natural movies while capturing hierarchical dependencies among them. Logistic regression is a natural choice for modeling a system with gaussian inputs (such as BOLD responses) and binary outputs (such as the presence or absence of a specific category). The most basic logistic regression approach would be to build a separate model for each category. However, this approach implicitly assumes that each category is independent from all the others. This assumption is clearly false when the categories are related hierarchically and it can lead to nonsensical results, such as decoding that a scene contains a *car* but not a *vehicle*.

**Figure 1:**
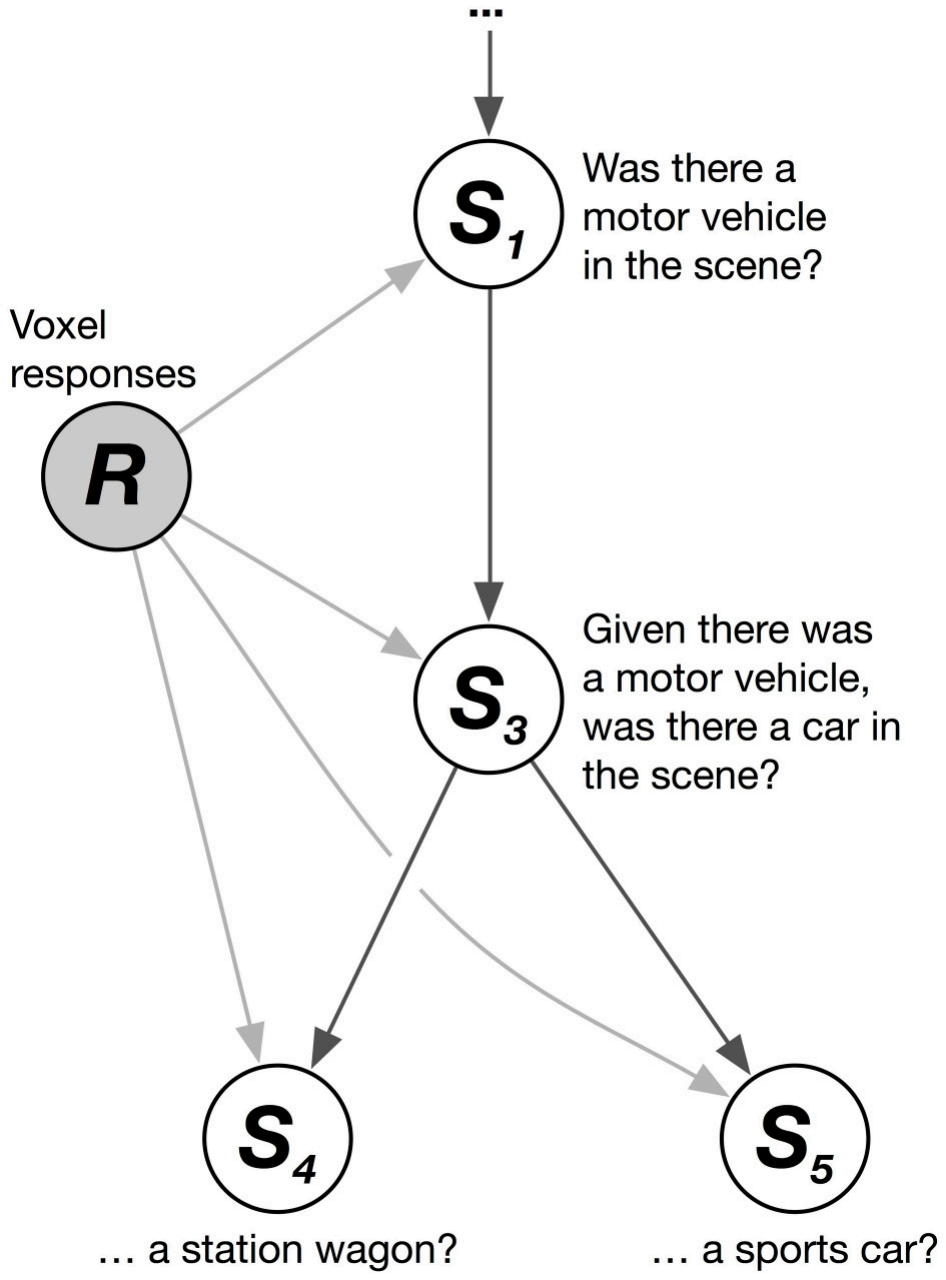
**Hierarchical logistic regression graphical model**. A hierarchical logistic regression (HLR) model was used to capture dependencies between decoded categories. A portion of the WordNet graph is shown here. White nodes represent categories to be decoded. The shaded node represents the observed voxel responses. The HLR model does not decode each category from the responses independently. Instead, it decodes the conditional probability that a hyponym (subordinate or child category) is present, given that its hypernyms (superordinate or parent categories) are present. The decoded probabilities of hypernyms and hyponyms are then multiplied together to compute the probability that the hyponym is present.

We solved this problem by combining multiple logistic regression models together hierarchically. The HLR model decodes the conditional probability that each category is present, given that its hypernyms (its superordinate or parent categories in the hierarchy) are present. These conditional probability relationships can be represented as a graphical model (Figure 1). The graphical model shows, for example, that the joint probability that a scene contains the categories *motor vehicle, car*, and *station wagon* (given a vector of brain responses, *R*) can be factorized into a product of conditional probabilities:

$$\begin{array}{l} P\left(motor\;vehicle,\;car,\;station\;wagon\;|R\right)\\ =P\left(motor\;vehicle\;|R\right)\times P\left(car\;|motor\;vehicle,\;R\right)\\ \;\times \;P\left(station\;wagon\;|car,\;R\right) \end{array}$$

Thus, the joint probability that a scene contains the categories *motor vehicle, car*, and *station wagon* is equal to the product of three conditional probabilities (note that this example is simplified; in our actual data *motor vehicle* is not a top-level category). Further, the marginal probability that the category *station wagon* is present in the scene is identical to this joint probability. This model does not treat each category independently. Instead, it assumes that each category is conditionally independent of the others, given its hypernyms. This structure enforces the sensible constraint that the probability of a *car* being in the scene is never greater than the probability of a *motor vehicle* being in the scene.

To estimate the full HLR model, we first estimated a separate logistic model for each conditional probability. Each logistic model predicts the binary presence or absence of a category given a vector of voxel responses across a few previous time points, *R*. Conditional probabilities were modeled by restricting the dataset that was used for model estimation. For example, to estimate a model for the conditional probability that a *car* is present given that a *motor vehicle* is present, we used only the time points when a *motor vehicle* was present (this technique has a side advantage of making model estimation much more efficient, since most of the conditional models are estimated using small subsets of the full dataset). The logistic models have a separate weight for each of the included voxels, at each time point. To account for hemodynamic lag, responses from multiple time points (4, 6, and 8 s after the stimulus being decoded) were also included.

To decode whether a category was present using the HLR models, we multiplied the conditional probabilities together. For example, to decode the probability that *car* was present at one time point, we first extracted the relevant voxel responses, then used the conditional logistic model to estimate the probability that *car* was present given that *motor vehicle* was present, and then used another conditional logistic model to estimate the probability that *motor vehicle* was present. Finally, we multiplied these probabilities together to find the joint probability that *car* and *motor vehicle* were present, given the voxel responses. It is clear from this formulation that the probability that *motor vehicle* is present can never exceed the joint probability that *motor vehicle* and *car* are present, thus respecting the hierarchical relationships between these categories.

**Figure 2:**
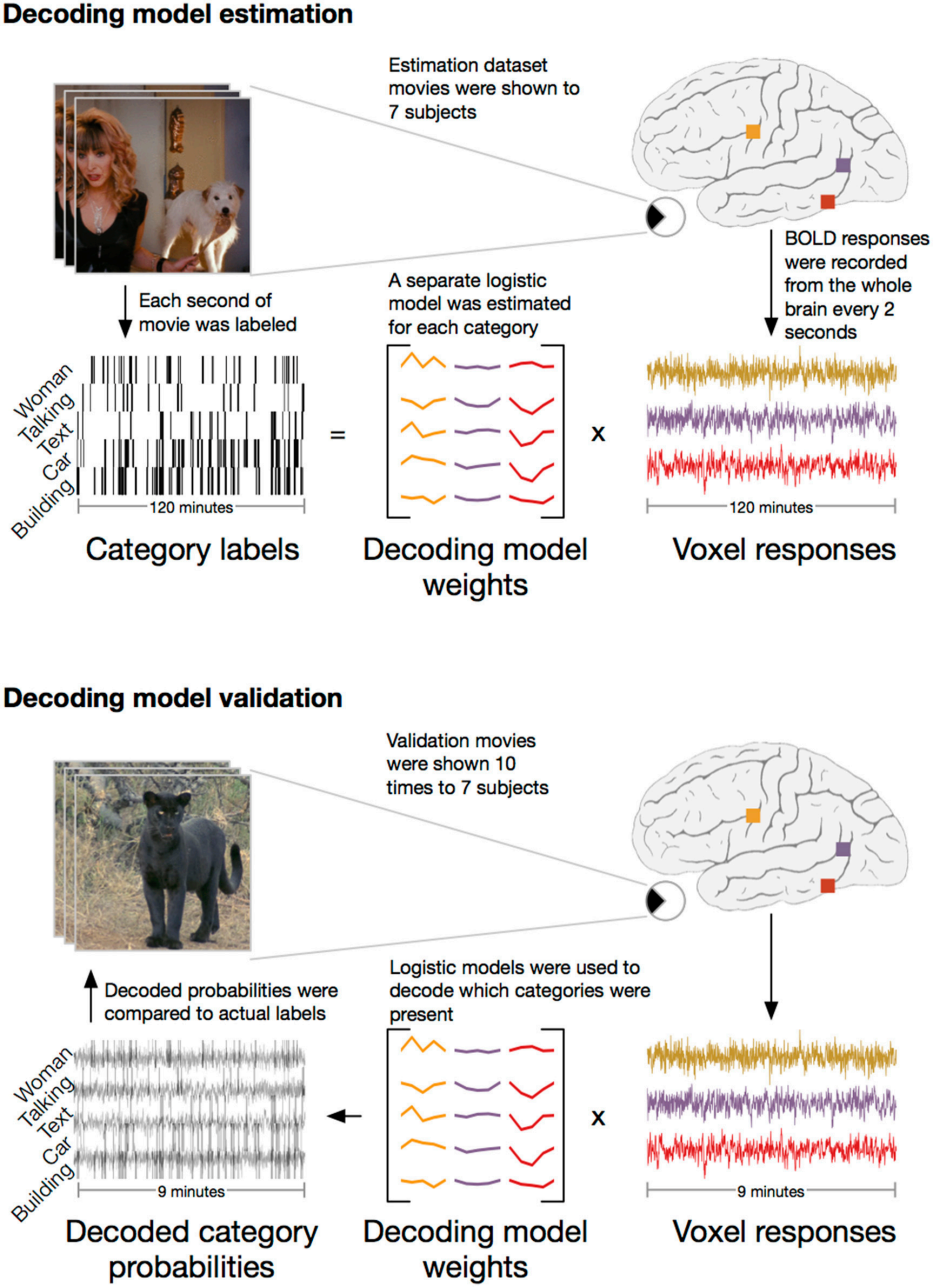
**Schematic of experiment**. The experiment consisted of two stages: **model estimation** and **model validation**. In the **model estimation** stage, seven subjects were shown 2 h of natural movies while BOLD responses were recorded using fMRI. Salient object and action categories were labeled in each 1 s segment of the movies. Direct decoding models were then estimated that optimally predicted the labels from linear combinations of voxel responses. In the **model validation** stage, the same seven subjects were shown 9 min of new natural movie stimuli that were not included in the estimation stimulus set. These movies were repeated ten times and the responses were averaged to reduce noise. The previously estimated models were then used to decode which categories were present in the movies. To assess model performance, the decoded category probabilities were compared to actual category labels in a separate validation set reserved for this purpose.

We applied the HLR modeling framework to BOLD fMRI responses recorded from seven subjects (Figure 2). First, fMRI responses were recorded while the subjects watched 2 h of natural movies. The WordNet (Miller, 1995) semantic taxonomy was used to label salient object and action categories in each one second segment of the movies. Using the 2 h of model estimation data, we then selected the 5000 voxels in each subject's cortex that had the most reliable category-related responses (see Methods for details). The category labels and BOLD responses for the 5000 selected voxels were then used to estimate a separate HLR model for each subject. To test the HLR models we recorded BOLD responses from the same subjects while they watched an additional 9 min of novel natural movies that had not been used to estimate the model. The model validation movies were repeated ten times and the responses were averaged across repeats to reduce noise. Finally, we used the HLR model for each subject to decode which categories were present in the validation movies.

## Materials and methods

### Subjects

Functional data were collected from seven human subjects. All subjects had no neurological disorders and had normal or corrected-to-normal vision. The experimental protocol was approved by the Committee for the Protection of Human Subjects at University of California, Berkeley. Written informed consent was obtained from all subjects. The data for five of the subjects used here were the same as those used in a previous publication (Huth et al., 2012).

### Experimental design

The stimuli for this experiment consisted of 129 min of natural movies drawn from movie trailers and other sources. These stimuli are identical to those used in earlier experiments from our laboratory (Nishimoto et al., 2011; Huth et al., 2012). WordNet was used to label salient objects and actions in each 1s segment of these movies (Huth et al., 2012). This resulted in 1364 unique labels. After adding entailed hypernym labels the total number of categories was 1705.

### MRI data collection and preprocessing

MRI data were collected on a 3T Siemens TIM Trio scanner at the UC Berkeley Brain Imaging Center, using a 32-channel Siemens volume coil. Functional scans were collected using a gradient echo-EPI sequence with repetition time (TR) = 2.0045 s, echo time (TE) = 31 ms, flip angle = 70 degrees, voxel size = 2.24 × 2.24 × 4.1 mm, matrix size = 100 × 100, and field of view = 224 × 224 mm. The entire cortex was sampled using 30–32 axial slices. A custom-modified bipolar water excitation radiofrequency (RF) pulse was used to avoid signal from fat.

Separate model estimation (fit) and model validation (test) datasets were collected from each subject in an interleaved fashion during three scanning sessions. The stimuli for the model estimation dataset consisted of 120 min of movie trailers. These stimuli are identical to the stimuli used in Nishimoto et al. (2011) and Huth et al. (2012), and are available for download from CRCNS: https://crcns.org/data-sets/vc/vim-2/about-vim-2. Functional data for the model estimation dataset were collected in 12 separate 10-min scans. The stimuli for the model validation dataset consisted of 9 min of movie trailers, repeated 10 times. Functional data for the model validation dataset were collected in 9 separate 10-min scans and then averaged. Note that the estimation and validation stimuli were completely distinct; no clips appeared in both sets. Throughout stimulus presentation for both datasets, subjects fixated on a dot that was superimposed on the movie and located at the center of the screen. The color of the dot changed four times per second to maintain visibility.

Each run was motion corrected using the FMRIB Linear Image Registration Tool (FLIRT) from FSL 4.2 (Jenkinson and Smith, 2001). A high quality template volume was then obtained by averaging all volumes in the run. FLIRT was also used to automatically align the template volume for each run to the overall template, which was chosen to be the template for the first functional movie run for each subject. These automatic alignments were manually checked and adjusted for accuracy. The cross-run transformation matrix was then concatenated to the motion-correction transformation matrices obtained using MCFLIRT, and the concatenated transformation was used to resample the original data directly into the overall template space.

For each voxel, low-frequency voxel response drift was identified using a median filter with a 120-s window and this was subtracted from the signal. The mean response of each voxel was then subtracted and the remaining response was scaled to unit variance.

Anatomical images were obtained using a T1 MP-RAGE pulse sequence. These images were then segmented to obtain a 3D representation of the cortical surface using Caret5 software (Van Essen et al., 2001).

### Model estimation

The HLR model includes a separate conditional logistic regression model for each category. Each conditional logistic regression model converts a spatiotemporal pattern of voxel activity to the binary presence (1) or absence (0) of one category, for time points where all of that category's hypernyms are present. While the cortex contains tens of thousands of voxels, many voxels are very noisy or contain little information about the stimuli. Thus, to reduce model complexity and reduce noise, only 5000 voxels in each subject were used as input to the HLR model. (Models were tested in one subject using 1000, 5000, and 10000 voxels. The best performance was found with 5000 voxels.) To find the best 5000 voxels for each subject, we first used regularized linear regression to estimate an independent encoding model for each voxel (encoding models predict the response of single voxels as a weighted sum across binary category labels). This modeling procedure was repeated 50 times, each time holding out and predicting responses on a separate segment of the model estimation dataset. Model prediction performance was then averaged across the 50-folds and the best 5000 voxels were selected. The model estimation dataset was used for this procedure, the validation data were reserved for use elsewhere.

For each scene, the spatiotemporal input to the HLR model is a length 15000 vector consisting of the BOLD responses for the 5000 selected voxels at three consecutive time points. Multiple time points were included because BOLD responses are slow, taking 5–15 s to rise and fall after a neural event (Boynton et al., 1996). Including multiple time points in the model allows the regression procedure to learn a linear filter that will deconvolve the slow BOLD response function from the stimulus time course. Thus to predict the presence of a category at time *t*, the model uses voxel responses at times *t*+*2, t*+*3*, and *t*+*4* TRs. With a TR of 2 s these delays correspond to 4, 6, and 8 s.

To build each conditional logistic regression model we used only the subset of the model estimation data where all the hypernyms of the selected category were present. For example, to build a model for the category *sports car* we selected all the time points where *car* was present. The model was then estimated using gradient descent with early stopping. First, the data were broken into two sets: 90% of the data were used for gradient descent and 10% were used to estimate the stopping point. At each iteration the weights were updated based on the gradient descent data, and then the model error was evaluated using the early stopping data. If the error on the early stopping data did not decrease for ten consecutive iterations, the gradient descent procedure was terminated. Voxel weights were initialized to zero and the bias term was set to produce the prior probability of the category given its hypernym (the prior probability was computed empirically across the training dataset). Each model was estimated three times using separate early stopping datasets and then the resulting weights were averaged.

We tested whether this gradient descent with early stopping produced different results from more standard L2-penalized regression, but found very little difference. We implemented L2 regularized logistic regression using scikit-learn (Pedregosa et al., 2011) with regularization coefficients ranging from 10^−6^ to 10^4^. For each of three bootstraps, we fit the model on 90% of the data and evaluated the loss on 10% to choose the best regularization coefficient. We then took the median regularization coefficient found over the bootstraps and used it to refit the model on the entire training set. We compared results of this procedure with those using the early stopping approach and found that, on average, regression with early stopping performed slightly better. Over all categories with AUC > 0.5 for either regression method, early stopping AUCs were on average higher by 0.09, and 59.0% of categories were better decoded by the early stopping model than L2 regularization. These differences appear to be due to early stopping doing much better on categories with few positive examples.

To avoid overfitting the model output was smoothed toward the original prior probability. We assumed a beta distributed prior on model outputs, with the mean set to the conditional prior probability for each category. We then fit a scaling parameter η such that $P^{*}\left(S_{i}|S_{\backslash i},R\right)=\frac{P\left(S_{i}|S_{\backslash i},R\right)+\eta P_{i,0}}{1+\eta }$ maximized the log likelihood of 1 min of held out data (where *P*(*S*_*i*_|*S*_\*i*_, *R*) is the output of the logistic model for the *i*th label given the other category labels and responses, and *P*_*i*, 0_ is the prior probability of seeing the *i*th label given that its hypernyms are present). This smoothed probability was used in all subsequent analyses.

All individual category models were then combined to form a HLR model that describes the full probability distribution over all scene labels.

### Model estimation with label noise

One potential issue with the logistic regression approach described above is that the manually assigned category labels in the model estimation dataset might be inaccurate or noisy. To account for this possibility we re-estimated logistic regression models for one subject using the method from (Bootkrajang and Kabán, 2012), which iteratively estimates a 2 × 2 label flipping probability matrix for each category, where the first row is the probability of getting a label of 0 or 1 given that the true label is 0 and the second row is the probability of getting a 0 or 1 given that the true label is 1. We re-estimated each logistic regression model twice: first initializing all the model weights to zero, and second initializing the model weights to the values found using our earlier logistic regression approach. In both cases we initialized the label error probability (i.e., the off-diagonal values in the label flipping matrix) to be 0.1. For both conditions, the flipping matrix rapidly converged to the identity matrix in nearly every category. The maximum estimated label error probability was 0.0086 (i.e., less than 1%). This suggests that the label flipping matrices for this experiment are virtually indistinguishable from the identity matrix. This is likely due to the fact that our stimuli were hand-labeled by a single individual rather than using a crowd-sourcing approach such as Amazon's Mechanical Turk.

### Model evaluation

#### Receiver operating characteristic (ROC) analysis

For each time point in the validation dataset we predicted the probability that each category was present in the stimulus using the HLR. Then an ROC analysis was used to assess model decoding performance for each category. To perform the ROC analysis we gradually increased a detection threshold from zero to one. For each threshold we computed the number of false positive detections (points where the predicted time course is higher than the threshold but the category is not present) and true positive detections (where the predicted time course is higher than the threshold and the category is actually present in the stimulus). Then we plotted the true positive rate (TPR) against the false positive rate (FPR) across all thresholds, producing the ROC curve.

A common statistic used to gauge detection performance is the area under the ROC curve (AUC). An AUC value of 1.0 represents perfect decoding, where the decoded probability for any time point where the category is actually present is higher than the decoded probability for every time point where the category is absent. We determined chance level of the AUC by shuffling the actual binary labels for each category across time. Blocks of four TRs were shuffled 1000 times to produce new time courses with the same prior probability and a similar autocorrelation structure to the original data (we tested other block sizes but found no difference in the results). The AUC was then computed for each of 1000 shuffled time courses, and the null distribution of AUCs was fit with a beta distribution centered at 0.5. Finally, we computed the probability of obtaining the actual AUC under this distribution. The actual AUC was declared significant if its probability under this null distribution was below the significance threshold. Significance thresholds were determined by applying the Benjamini-Hochberg procedure (Benjamini and Hochberg, 1995) to limit the false discovery rate, *q(FDR)*, across multiple comparisons to 0.01.

#### Model likelihoods

The ROC analysis tests how well each category is decoded across all time. Yet it is also important to test how well all the categories are decoded within each time point. To test this we calculated the likelihood of the actual category labels at each time point, given the decoded category probabilities. This likelihood was computed as the product of the probabilities of obtaining the actual binary label for each category under the model. For the null model we used the prior probability according to the model estimation dataset, which was constant over time. We then quantified model performance as the relative log likelihood ratio between the HLR model and the null model. To estimate chance level performance we shuffled the model output for each category across time 100,000 times, recomputing the log likelihood ratio on each shuffle. The relative log likelihood was declared significant if the probability under the shuffled distribution was below the significance threshold (*p* < *0.01*).

## Results

### Decoding performance for individual categories

**Figure 3:**
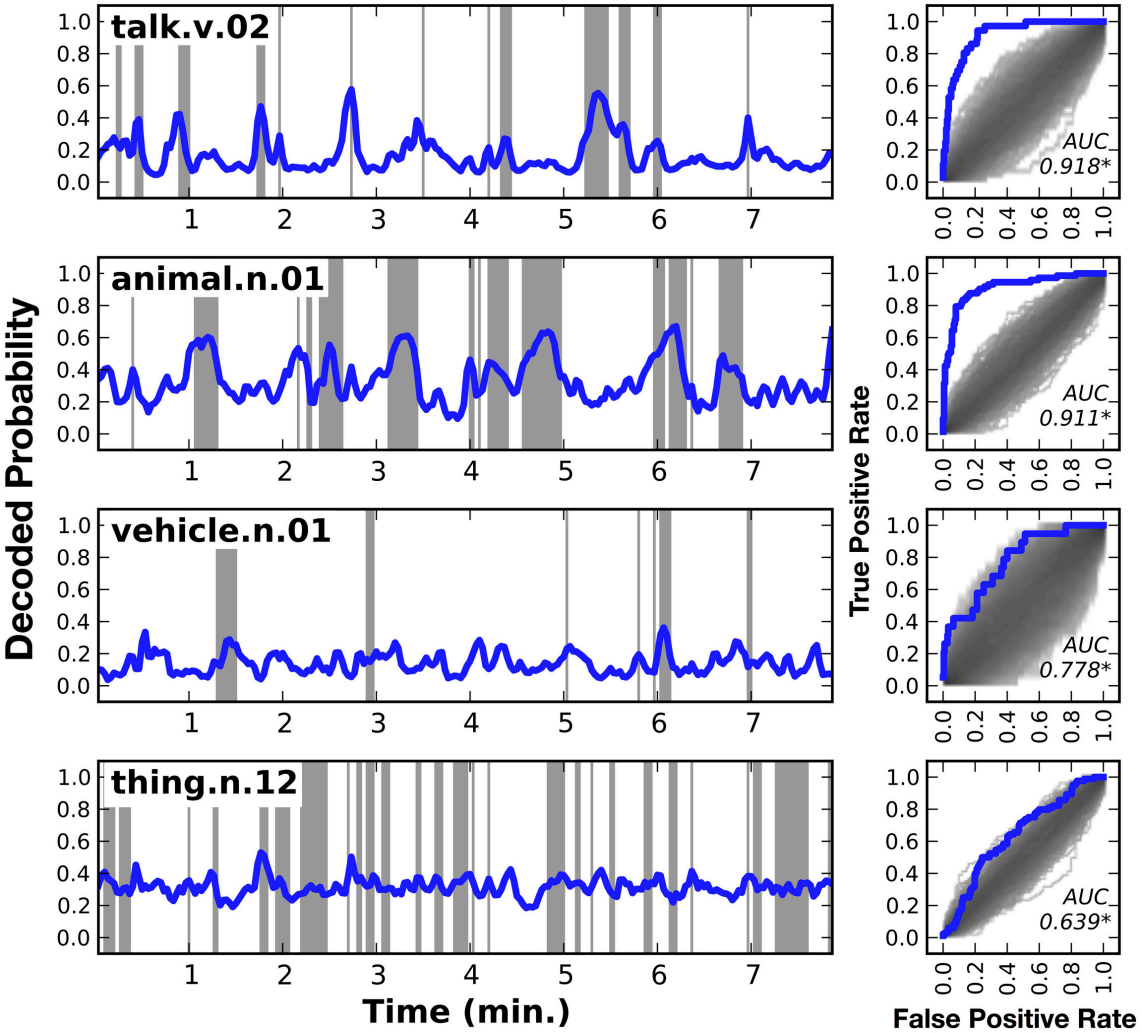
**Decoded time courses and decoding performance from one subject for four individual categories**. Results for four out of the 479 categories decoded in this study. (**Left**) Each row gives the decoded probability that a specific category of object or action was present in the movie over time. Blue lines show the decoded probability and gray regions show time points when the category was actually present in the movie. Decoded probabilities for the verb *talk* and the noun *animal* are high when those categories are present, and lower at other times. However, the probabilities are not temporally precise. For example, at 2.7 min into the movie *talk* appears for a single time point, but the decoded probability takes several time points to rise and fall. Decoded probabilities for the noun *vehicle* and the noun *thing* (specifically *thing.n.12*, which includes categories as diverse as *body of water* and *body part*) are less accurate than *talk* or *animal*. However, the decoding model correctly assigns low confidence to its predictions, as evidenced by the fact that the decoded probability for *thing* hovers around the prior value of 0.32. (**Right**) Receiver operating characteristic (ROC) analysis summarizing overall decoding accuracy for each of the four categories. The ROC plots the true positive rate (TPR) as a function of the false positive rate (FPR) of the decoder. Performance of the decoder is shown in blue. Chance performance was determined by shuffling the stimulus timecourse and recomputing the ROC curve (see Methods). The distribution of curves across 1000 shuffles is shown on the same plot in gray. The area under the ROC curve (AUC) is shown within each panel and significant values (q(FDR) < 0.01) are marked with an asterisk. The ROC curves indicate that both *talk* and *animal* are decoded accurately, but *vehicle* and *thing* are not decoded particularly well. Similar plots for the other six subjects are shown in Supplementary Figures 1–7.

Figure 3 shows HLR model decoding performance in one subject for a few different categories: *talk, animal, vehicle*, and *thing* (similar plots for the other six subjects are shown in Supplementary Figures 1–7). Panels on the left side of the figure show the decoded category time course, across the model validation dataset. Shaded regions indicate periods when the category was actually present. Panels on the right side of the figure show the receiver operating characteristic (ROC) curve for the corresponding category decoder. The shaded region under the ROC curves shows the density of the null distribution of ROC curves, which was determined by shuffling. All the AUCs shown in this figure are significantly greater than expected by chance (q(FDR) < 0.01).

The first row of Figure 3 shows the decoded time course for the verb *talk*. The decoded probabilities are very high when *talk* occurs in the movie and they are relatively low at other times. There are no false positive peaks in the decoded time course. However, the decoded time course is not temporally precise: it takes a few seconds to rise and fall. For example, at 2.7 min into the movie *talk* appears for a single time point, but the decoded probability begins to rise several time points earlier and then takes several time points to fall back to baseline after the category disappears. This temporal imprecision appears even though the HLR model includes responses from multiple time lags, which should partially compensate for the sluggish hemodynamic response. This might be because the HLR model decodes the categories at each time point independently, and does not consider the categories decoded for other time points. Nevertheless, the area under the ROC curve (AUC) is 0.918, demonstrating that the decoder is extremely accurate. This suggests that the cortical representation of *talk* is sufficiently robust to be decoded reliably using fMRI.

The second row of Figure 3 shows the decoded time course for the category *animal*. The AUC for *animal* is 0.911, again indicating that the decoder is extremely accurate. As with *talk*, this suggests that the cortical representation of *animal* is sufficiently robust to be decoded reliably using fMRI.

The third row of Figure 3 shows the decoded time course for the category *vehicle* (This is a general category that includes several more specific categories such as *car, motorcycle*, and *boat*). The decoded time course is very high at 6.1 min, when *vehicle* is actually present in the stimulus. However, the decoded time course was low during several other periods when *vehicle* was present. At other times, such as 0.5 min, the decoded time course is high but no *vehicle* is present. In this case the AUC is 0.758, indicating that the overall accuracy of the decoder is fair. This suggests that the cortical representation of *vehicle* is not as reliable or distinctive as the representations of *talk* or *animal*.

The fourth row of Figure 3 shows the decoded time course for the category *thing*. *Thing* (specifically *thing.n.12* in WordNet) is a high-level category that includes categories such as *body part* and *body of water*. The decoded time course is consistently intermediate, and there are few times where the decoded probability was very high or very low. Time points where a *thing* was actually present in the stimulus have only marginally higher decoded probabilities than time points where *thing* was not present. The AUC of 0.694 is statistically significant, but it is much lower than the AUC obtained for other categories shown here. This suggests that the cortical representation of *thing* is not distinctive as is the representations of other, more specific categories. We believe that this is because *thing* is an artificial category invented by WordNet that is not strongly represented in the brain.

### Decoding performance for all categories

**Figure 4:**
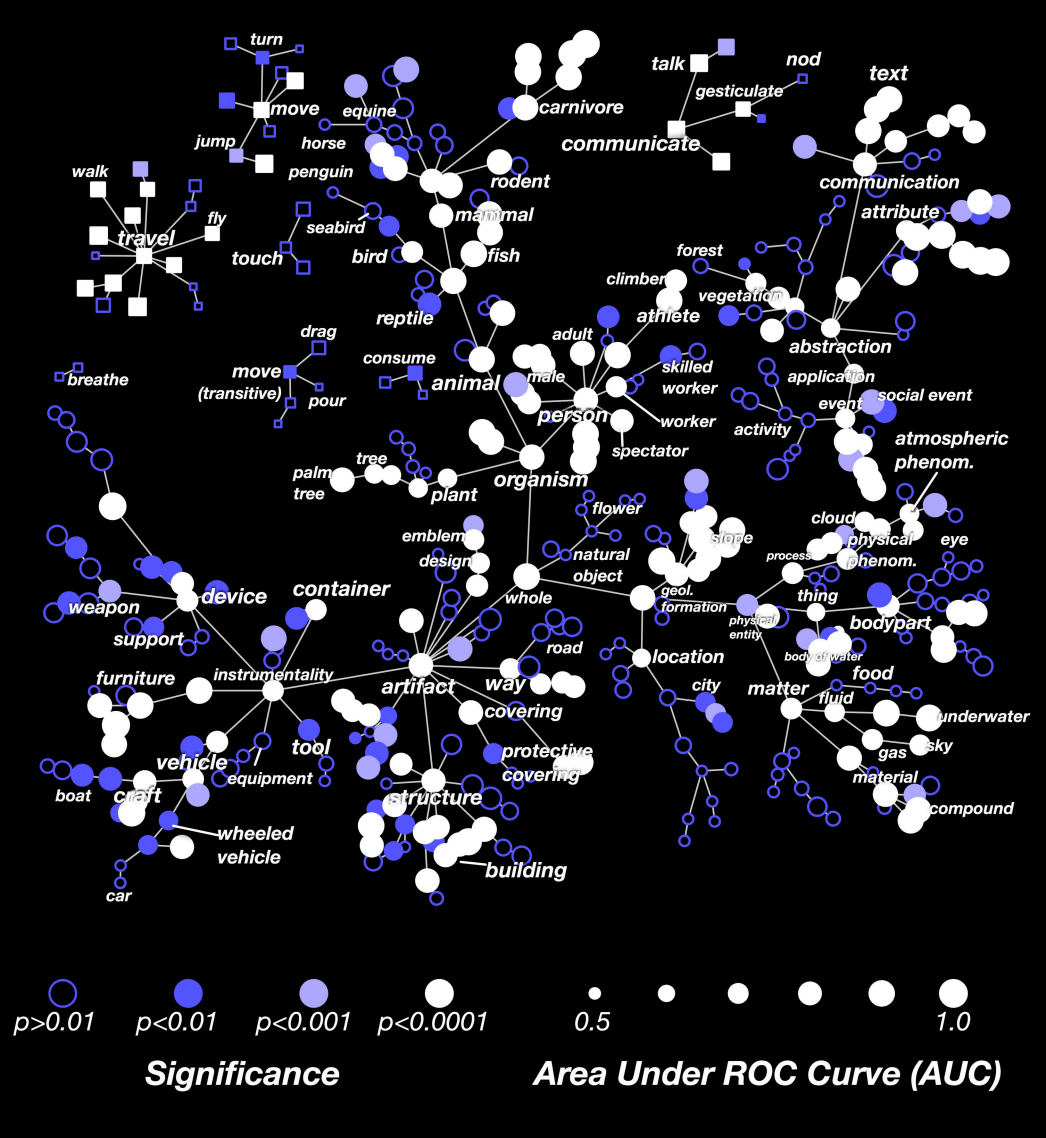
**Graphical visualization of decoding accuracy**. The plot is arranged according to the graphical structure of WordNet. Circles and squares denote the 479 categories that appeared in the movies used for model validation. Circles indicate objects (nouns) and squares indicate actions (verbs). Decoding performance was aggregated across all subjects by concatenating the decoded probability time courses. The size of each marker denotes the area under the ROC curve (AUC) for that category, ranging from 0.5 to 1.0. The marker colors denote the *p*-value for that category's AUC; deeper blue reflects larger *p*-values. Categories where decoding accuracy is significant are displayed as filled circles (q(FDR) < 0.01). The AUC is high for some general categories, such as *person, mammal*, and *artifact*. It is low for others, such as *thing, matter, instrumentality*, and *abstraction*. This suggests that some general categories are well represented in the brain at a scale that can be measured using fMRI, while others are not. The AUC is usually low for specific categories that are more infrequent. This doesn't necessarily imply that infrequent categories are represented poorly in the brain; it may merely reflect insufficient data. The AUC is also low for background categories, such as *plant, location*, and *atmospheric phenomenon*. This may occur because subjects do not usually attend to these categories well unless instructed to do so explicitly. Similar plots for each subject separately are shown in Supplementary Figures 1–7.

The results in Figure 3 showed that the decoder is not equally successful for all categories. To explore this issue further, we computed the decoding performance (AUC) for all categories that appeared in at least 3 time points in the validation dataset. In Figure 4 we show these AUCs in a graph that is organized according to the structure of the WordNet semantic taxonomy (similar plots for each subject separately are shown in Supplementary Figures 1–7; the 30 best-decoded categories across all subjects are listed in Supplementary Table 1). Here the color of each node reflects the AUC (integrated across all the subjects), and the saturation reflects confidence in the AUC estimate.

Many general categories, such as *person, mammal, communicate*, and *structure* were decoded accurately, suggesting that these categories are represented by specific, consistent patterns of activity in the brain. In contrast, other general categories, such as *thing* and *abstraction*, were decoded poorly, even though we can accurately decode the hyponyms (or subordinate categories) of these poorly decoded general categories. For example, *thing* is poorly decoded, but its hyponyms *body of water* and *body part* are both decoded accurately. This suggests that *body part* and *body of water* are represented very differently, so the linear model cannot decode both categories simultaneously. Among actions (shown as square markers) we found that communication verbs, travel verbs, and intransitive movements (e.g., *jump, turn*) were usually decoded significantly and accurately, while consumption verbs and transitive movements (e.g., *drag, pour*) were usually decoded poorly.

### Conditional decoding performance

**Figure 5:**
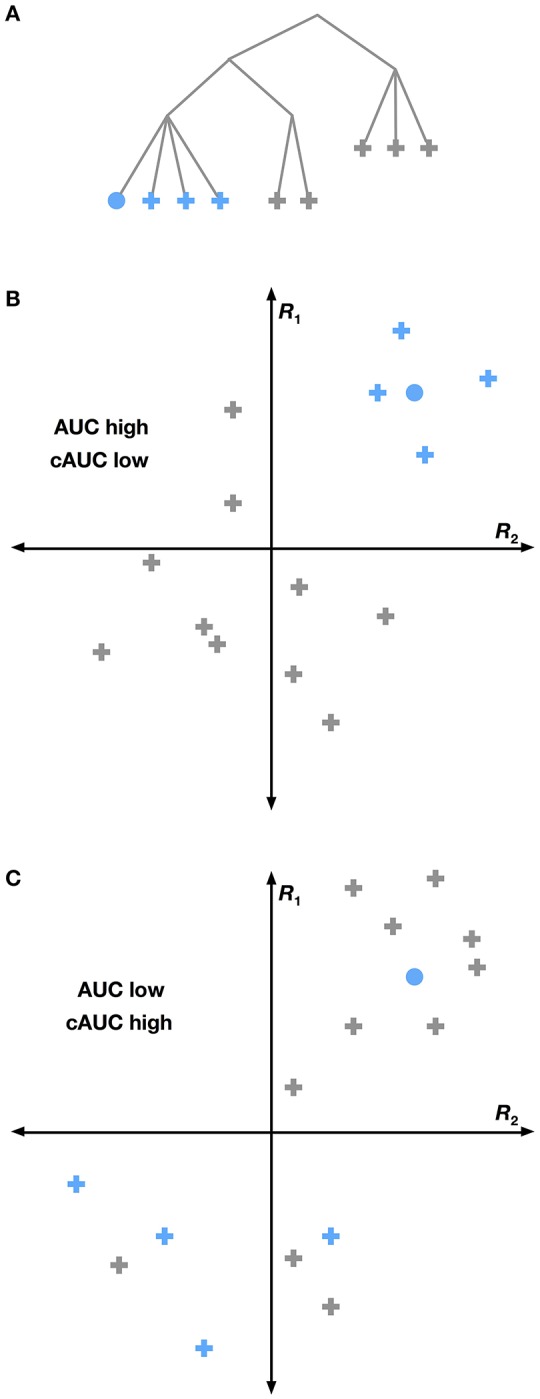
**Conditional versus full AUC**. The HLR assumes that the structure of WordNet is reflected in the brain. Yet this may not be the case. If a particular grouping of categories (shown as blue nodes in **A**) is strongly reflected in the brain, then we would expect that the group will be highly separable from all other categories. This situation is shown graphically in **(B)**, where voxel responses to several categories are plotted in a hypothetical 2-dimensional response space. Categories within a group are shown in blue and other categories are gray. The voxel response to the category we are trying to decode is shown as a circle. Here the blue categories are easily linearly separated from other categories, leading to a high total AUC for the selected category. A different situation is shown in **(C)**, where the grouped categories do not elicit very similar responses. Here the selected category is easier to distinguish from its siblings than from other categories, leading to a high cAUC and lower total AUC. This suggests that for WordNet-based category groupings that are not reflected in the brain, the cAUC will be significantly higher than the total AUC.

The HLR approach assumes that cortical responses follow the WordNet taxonomy, but this assumption is likely false in some cases. Therefore we performed an analysis that shows which hypernymy relationships in WordNet were not reflected in brain activity. Under the HLR approach we used WordNet to construct conditional models that decode the presence of a given category including all of its hyponyms. For example, the conditional model for *car* must distinguish between any car (e.g., *station wagon, sports car*, etc.) and any other *motor vehicle*. These models implicitly assume that all the hyponyms of any given category elicit similar responses in cortex (Figure 5). If this assumption is true, then overall decoding performance will be good, but it may be difficult to distinguish between the hyponym categories. If this assumption is false, then overall decoding performance will be poor, but it will be easy to distinguish between hyponyms.

We used this logic to construct a test for each hypernymy relationship in the subset of WordNet used in this study. For each category, we computed the conditional AUC (cAUC) using only the time points in the validation dataset when all the hypernyms of that category were present. Thus the cAUC shows how well a category can be distinguished from its siblings. We then compared the cAUC to the overall AUC for each category. If the cAUC was significantly higher than the overall AUC, then we concluded that the assumed relationship between this category and its hypernym is not reflected in brain activity.

**Figure 6:**
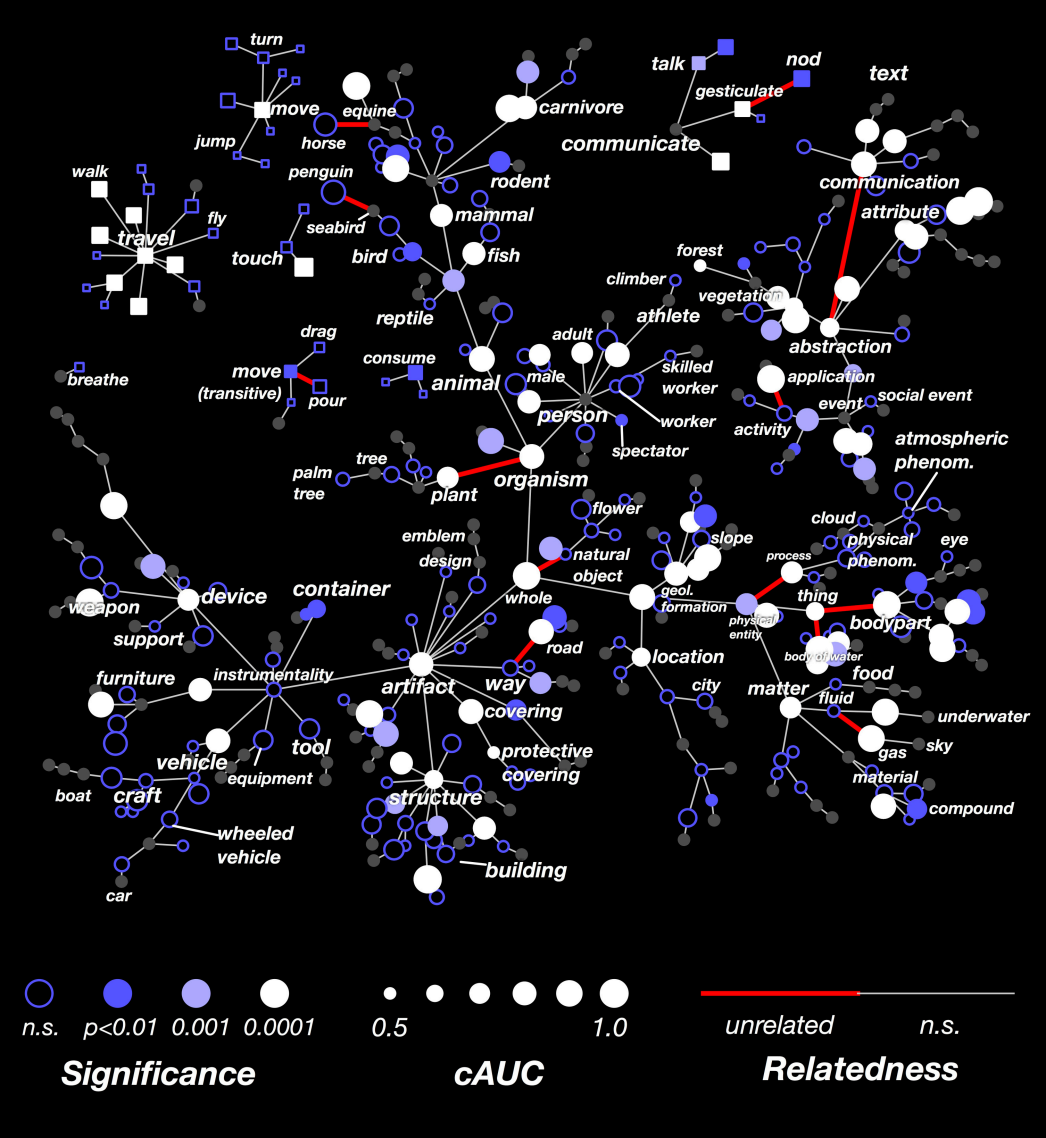
**Graphical visualization of decoding accuracy after conditioning on parent categories**. Decoding performance for each of the 479 categories, conditioned on the presence of their hypernym in the scene, concatenated across subjects. The figure is arranged identically to Figure 4. The conditional AUC (cAUC) was computed only on time points where the hypernyms of a category are present, which forces the model to discriminate amongst sibling categories. If the cAUC for a category is greater than the full AUC, it means that the category is easier to distinguish from its siblings than from other categories. The significance of this difference was evaluated for each edge in the WordNet graph. If a category is significantly less similar to its siblings than would be expected by chance, the edge is colored red. Thus WordNet links between categories that are unrelated in the brain appear red. Edges between *thing* and its hyponyms *body of water* and *body part* appear red because these categories are not represented similarly in the brain. Also, the edge between *organism* and *plant* appears red, likely because *plant* is the only non-animate hyponym of *organism*. Categories for which the conditional entropy is too low to reliably estimate cAUC are colored gray.

The results of this analysis are shown in Figure 6. Here the cAUC for each category is plotted on the same WordNet graph used in Figure 4. The size and color of each node reflects the cAUC of the corresponding category. For categories where the cAUC is significantly higher than the overall AUC, the edge linking that category to its hypernym is colored red (all significant relationships are listed in Supplementary Table 2). In total, we found that 17 of the relationships given by WordNet were significantly inconsistent with brain responses. Several of the significantly inconsistent relationships highlight categories that are technically related, but which are very different from their siblings. For example, *plant* is the only non-animate branch of *organism, horse* is the only equine ridden by humans, and *penguin* is the only flightless *seabird*. Other significantly inconsistent relationships appear between very high-level categories, likely reflecting difficult choices made in the design of WordNet. For example, the relationships between *thing* (specifically *thing.n.12*) and its hyponyms *body part* and *body of water* seem artificial. In sum, these results show several category relationships that should be reconsidered if WordNet is to be used to further model brain responses.

### Decoding performance for individual time points

The HLR model recovers information about the presence of individual categories in the stimulus, but in natural movies many different categories appear at each point in time. To test how well the HLR model decodes all of the categories present at each time point, we computed the probability of the actual categories present in the stimuli, *S*(*t*), given the model estimates, $\theta \left(t\right)=\hat{P}\left(S\left(t\right)|R\right)$. In order to compare to the level of performance that would be expected by chance, we normalized this value by the prior probability of the actual categories, *P*_0_(*S*). Here we approximated *P*_0_(*S*_*i*_) by setting it equal to the proportion of the time that the category *S*_*i*_ was present in the movies used for model parameter estimation. Thus, the likelihood of the decoded category relative to the prior for each time point is given by:

$$\begin{array}{l} \frac{P\left(S\left(t\right)|\theta \left(t\right)\right)}{P_{0}\left(S\right)} \end{array}$$

**Figure 7:**
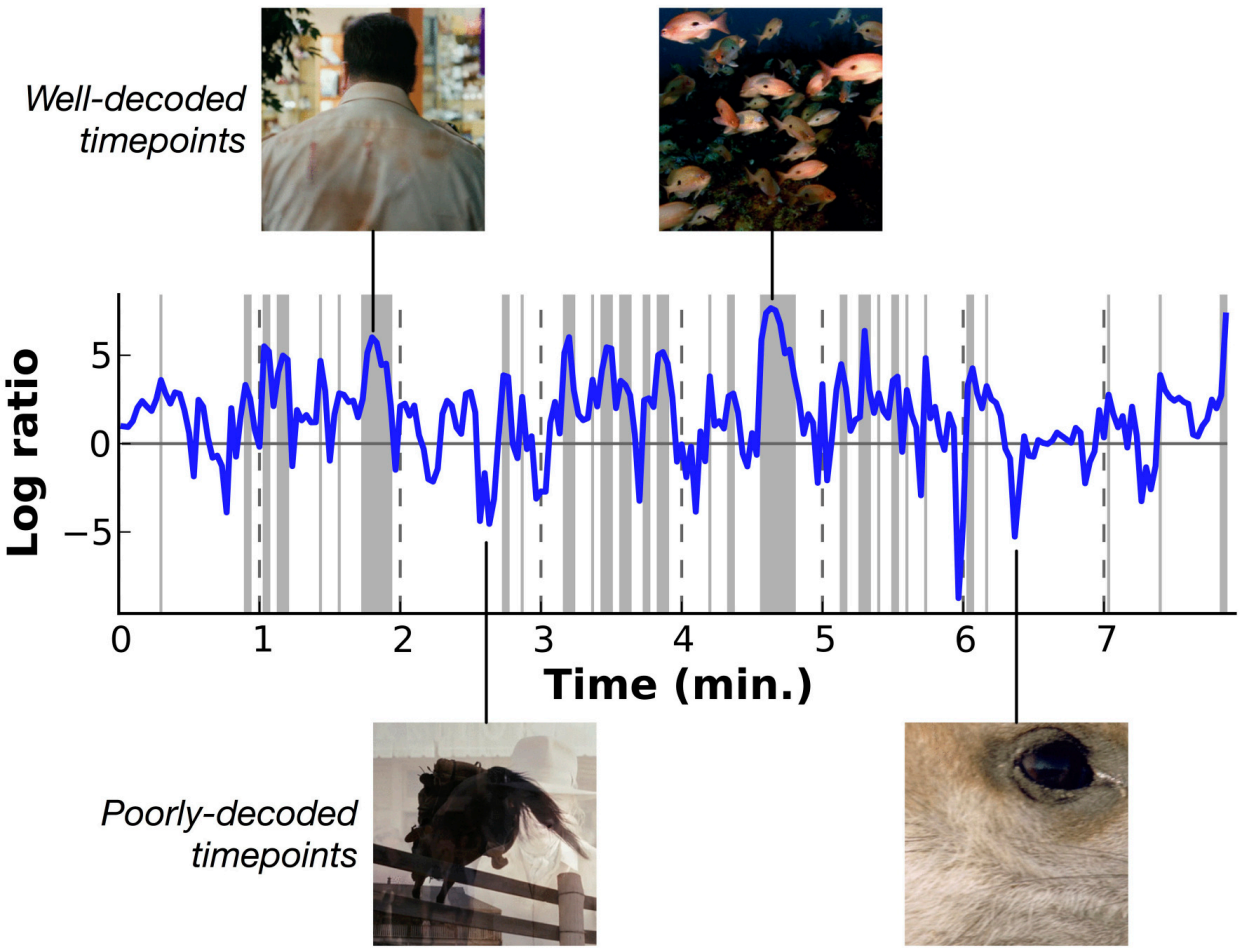
**Overall decoding performance at each time point across all object and action categories**. Here results at each time point have been averaged across all five subjects. Decoding accuracy is expressed as the log likelihood of the actual category labels given the model, relative to the prior likelihood that each category is present. Values at zero indicate that the model performs as well as would be expected by merely guessing based on prior probabilities. Shaded regions indicate performance significantly better than chance (*p* < 0.01 uncorrected, permutation test). Two examples of well-decoded time points are shown at the top. One is a man walking, the other is an underwater scene showing a school of fish. These are simple and stereotypical scenes that can be decoded accurately. Two examples of poorly-decoded time points are shown at the bottom. One is mid-fade transition between scenes of a horse jumping and a woman drinking, the other is a closeup of a deer's eye. The transition scene cannot be decoded accurately because the temporal precision of the decoder is poor. The deer eye is an atypical scene that is found rarely in the stimuli used to estimate the voxel-wise models. Similar plots showing data for each subject separately are shown in Supplementary Figures 1–7.

Figure 7 shows the relative log likelihood across time, averaged across subjects (similar plots showing data for each subject separately are shown in Supplementary Figures 1–7). Log likelihood ratios greater than zero indicate periods when HLR model estimates are relatively more likely than the prior, and log ratios less than zero indicate periods when the model is relatively less likely than the prior. This figure shows that some periods in the movie are decoded consistently better than others. Examination of the stimuli that appeared during the peaks and troughs in decoding performance shows that decoding is most accurate for underwater scenes and for scenes that contain a single person. These scenes contain only a few categories, all of which are well-modeled by the decoder. We observe a weak trend toward lower relative log ratios when the number of categories in a scene is greater (see Supplementary Figure 8), and decoding is relatively poor for scenes that contain unusual categories (such as close-up scene of wine being poured into a wineglass) and for time points that contain transitions between scenes.

### Comparison of original movies with decoded categories

To provide an intuitive and accessible demonstration of the performance of the HLR decoder, we constructed a composite video that shows the stimulus movie on the left, and the categories with the highest decoded probability on the right (see Supplementary Video 1). The size of each label corresponds to the predicted probability that the category is present. Note that the stimuli shown here are from the model validation set, and were not used to train the decoder. This demonstration shows that the decoder successfully recovers information about many categories regardless of the specific content of the movie.

### Mapping decoding model weights across the cortex

Since the HLR decoder seems able to recover many object and action categories from BOLD responses, one might naturally ask which voxels are used to decode each category. However, note that decoding results must be interpreted with caution; asking which voxels contribute to decoding is not equivalent to asking which voxels represent information about a category (Haufe et al., 2014; Weichwald et al., 2015). Voxels that have small (or zero) decoding weights for a category may still represent information about that category, but if the voxel also represents information about other categories then it might not be particularly useful for decoding. Conversely, voxels that have large decoding weights may not represent information about a category, but might instead be correlated (or anti-correlated) with noise in voxels that do represent that category. These interpretational issues are much less serious for encoding models (Huth et al., 2012), which predict responses from stimuli rather than predicting stimuli from responses. Voxels that have small encoding model weights for a category are likely not involved in representing that category. Voxels that have large encoding model weights either respond directly to the category or to some aspect of the stimulus that is correlated with the category. For these reasons, we direct readers who are interested in how these categories are represented across the cortex to our encoding model study that used the same dataset as the one analyzed here (Huth et al., 2012).

**Figure 8:**
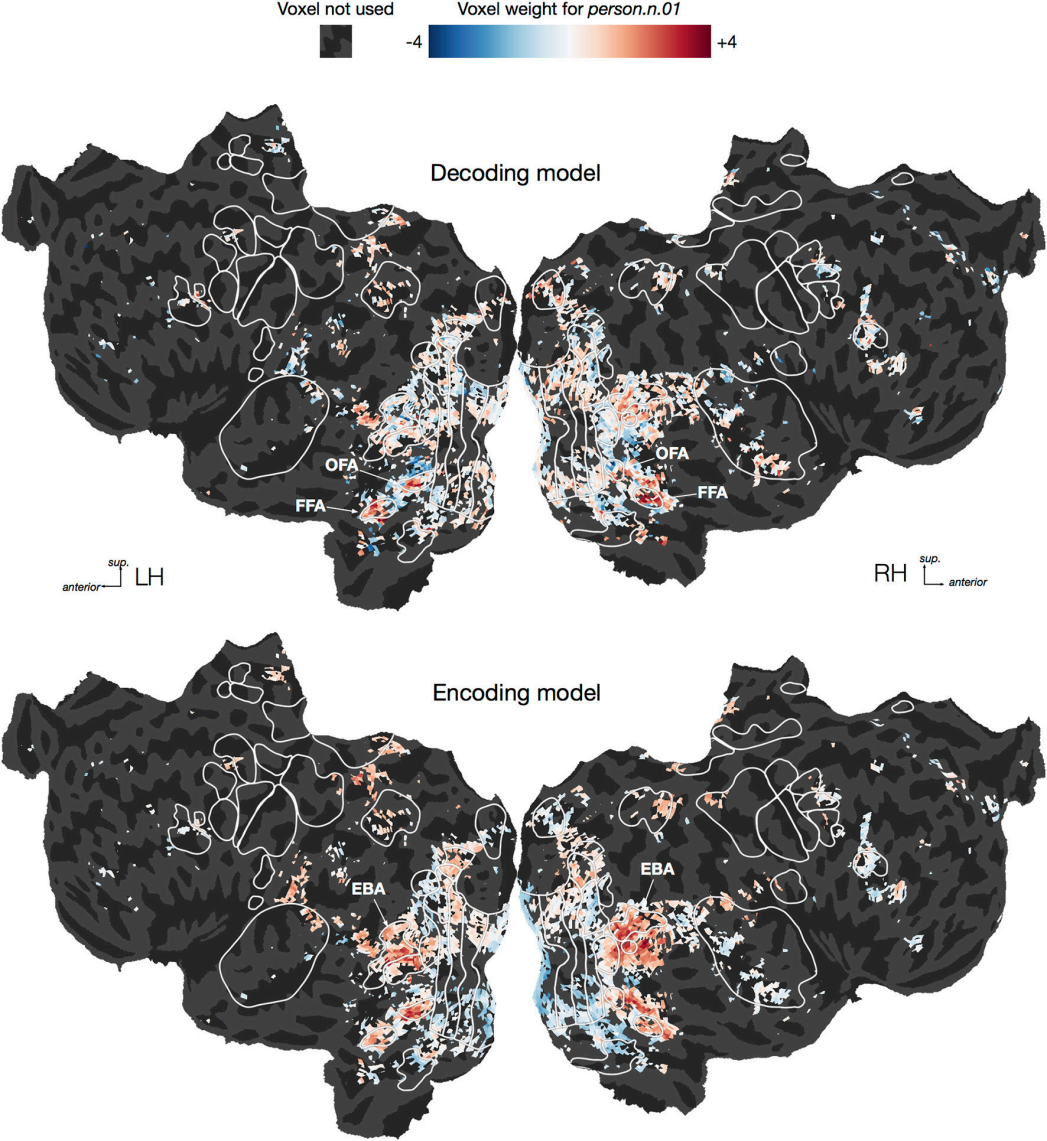
**Flattened cortical maps showing example decoding and encoding model weights for one category**. We plotted both decoding and encoding model weights for one category, *person.n.01*, in one subject. For the purpose of this visualization we constructed a direct logistic decoding model for this category (i.e., we did not condition on its hypernyms). We averaged the decoding weights across the three delays within each of the 5000 voxels, and then rescaled the resulting average weights to have a standard deviation of 1.0. Similarly, we averaged the encoding weights for the same 5000 voxels across the three delays and then rescaled the results. For the decoding model, we see large positive weights in the occipital face area (OFA) and fusiform face area (FFA), suggesting that activity in those regions predicts the presence of a person in a visual scene. For the encoding model, we also see positive weights in OFA and FFA, but some of the most positive weights appear in the extrastriate body area (EBA). This suggests that the presence of a person in a visual scene predicts EBA responses. However, the lack of large EBA weights in the decoding model suggests that EBA responses are not specific to seeing a person. This illustrates the inherent difficulty of interpreting weights from a decoding model.

To illustrate the difficulty of interpreting decoding model weights, we plotted both decoding and encoding weights for one category, *person.n.01*, on cortical flat maps for one subject (Figure 8, For illustrative purposes this decoding model was fit using the entire dataset rather than only the time points containing the hypernyms of *person*). Earlier studies have shown that several brain areas respond selectively to human faces and bodies, including the fusiform and occipital face areas [FFA Kanwisher et al., 1997 & OFA Kanwisher et al., 1997; Halgren et al., 1999] and the extrastriate body area (EBA Downing et al., 2001). Therefore one might naïvely expect that voxels in all of those areas would be assigned large positive weights in the decoding model for *person*. However, the decoding model only has high weights in the face areas (FFA and OFA), but not the body area (EBA). Thus, interpreting the decoding model weights directly would lead to the conclusion that EBA does not represent information about humans. In contrast, the encoding model has high weights in EBA as well as the face areas, demonstrating that EBA does, as expected, respond to the presence of humans. So why was EBA ignored by the decoding model? One possibility is suggested by our earlier encoding model study, which showed that EBA responds to both animals and humans, but that FFA and OFA are both relatively more selective for human faces (Huth et al., 2012). Based on these encoding model results, the conclusion from the decoding model—that EBA does not represent information about humans—appears false. Instead, we should conclude that EBA represents information about humans in addition to other categories. This example illustrates that directly interpreting decoding weights can easily lead to erroneous conclusions, and should thus be avoided whenever possible. Instead, questions about cortical representation should be answered using encoding approaches.

## Discussion

In this study we showed that it is possible to accurately decode the presence or absence of many object and action categories in natural movies from BOLD signals measured using fMRI. These include general categories, such as *animal* and *structure*, specific categories, such as *canine* and *wall*, and actions, such as *talk* and *run*. However, decoding accuracy was better for some categories than it was for others. In particular, we found that decoding accuracy was generally better for scenes that contained relatively fewer categories than for scenes containing relatively more categories. This suggests that the amount of category-related information available in BOLD signals at each timepoint is limited.

Our decoder used a HLR model based on the graphical structure of WordNet, a semantic taxonomy that was manually constructed by a team of linguists (Miller, 1995). This hierarchical approach has two important features that make it attractive for decoding categories from natural stimuli. First, it provides a means to decode information at many different levels of detail simultaneously. This is vital for decoding natural stimuli, where it is unclear what level of detail should be used to describe any particular object or action. For example, the same object could be correctly labeled as a *vehicle, car, sports car*, or *Ford Mustang*. By decoding all levels of detail simultaneously the HLR model sidesteps the question of which level is most appropriate: a scene has some probability of containing a *vehicle*, some probability of containing a *car*, and so on. This allows the HLR model to decode relatively more general categories when specific categories occur infrequently or when they are difficult to distinguish using the brain data. For example, the HLR could show that it is difficult to decode the specific category *Ford Mustang* but easy to decode the general category *vehicle*. Using multiple levels of detail also allows the HLR to generalize to new categories: even if *Ford Mustang* did not appear in the model estimation dataset, the HLR might decode the presence of *car* based on earlier examples of that category.

The second important feature of the HLR model is that it uses the relationships between categories to rationally constrain the decoding results. If these constrains were not included, simultaneously decoding hierarchically related categories could easily lead to nonsensical results. For example, a naïve simultaneous decoder might find that the probability of a particular scene containing a *car* is higher than the probability of that scene containing a *vehicle*. This would be impossible, since every *car* is also a *vehicle*. The HLR avoids this issue by constraining the decoded probability of any category to be at most equal to the decoded probability of that category's hypernyms in WordNet. This approach builds on an idea known as “structured output” or “hierarchical learning” (DeCoro et al., 2007; Silla and Freitas, 2011), a field of machine learning concerned with problems where the output is known to have some specific statistical structure. In the decoding problem that we are addressing here, the structure of the output is defined by the WordNet category hierarchy and the knowledge that a category can never be present unless its hypernyms are present. This information is incorporated into the model by using what is known as a “local” or “siblings” policy for selecting the negative training examples for each category (Wiener et al., 1995; Silla and Freitas, 2011). This nomenclature comes from the fact that the negative examples for each category are chosen to be the time points where the category's siblings are present (and thus the category's parent is present), but where the category itself is not. This approach is also able to make model estimation more computationally efficient without decreasing performance because it only uses relevant training examples (Fagni and Sebastiani, 2007).

One potential issue with the HLR approach is its implicit assumption that all the hyponyms of a category elicit similar brain responses. This could lead to problems because the category relationships come from WordNet, which is a hand-constructed semantic taxonomy and thus is not guaranteed to reflect brain activity. To address this issue, we tested each of the relationships specified in the subset of WordNet spanned by our stimuli. This was done by examining how easily each category could be distinguished from its siblings under the same hypernym.

We found that two specific types of WordNet relationships were not reflected in cortical representations. The first are relationships that are technically correct, but where the specific category fails to share many features with the general category. For example, the relationship between *plant* and *organism* was not reflected in brain activity, likely because *plant* is the only inanimate hyponym of *organism*. The second type are relationships that seem abstrusely academic and may be idiosyncratic to WordNet. For example, the relationships between *thing.n.12* and its hyponyms *body part* and *body of water* were not reflected in brain activity, likely due to the fact that *body part* and *body of water* are not similar categories by most metrics. It is possible that modifying the WordNet hierarchy by removing or changing these poorly represented relationships would actually improve decoding performance. Modifying WordNet based on brain data might also prove useful for understanding how categories are represented in the brain. Future studies might even replace WordNet with a hierarchy learned entirely from brain data. Efforts to construct category hierarchies directly from brain data have already yielded plausible results for a few categories (Kriegeskorte et al., 2008).

One alternative to the HLR approach would be to decode only the “basic level” categories (Rosch et al., 1976). This would simplify some aspects of the modeling, since it would obviate the need to account for relationships between categories. Furthermore, basic level categories might be better represented in cortex than superordinate or subordinate categories (Iordan et al., 2015). However, a basic-level decoder would not be as powerful as the HLR decoder. First, the basic level category of a particular object is highly dependent on context (Rosch, 1978). For example, observers might agree that the basic level category for a specific object in a city scene is *car*, but the same object seen in a car dealership might be called *sports car*. It is not clear that estimating separate decoding models for *car* and *sports car* would make sense in this situation. Second, it would be impossible for a basic level category decoder to generalize to new categories. For example, suppose that several scenes in the validation dataset contained *trains*, but that trains did not appar in the estimation dataset. While neither the HLR model nor a basic level decoder would be able to directly decode the presence of *train*, the HLR model might be able to decode the presence of *vehicle* based on other examples such as cars, boats, and airplanes.

Another alternative to the HLR model would be to represent categories not as binary variables, but as vectors of features, topic probabilities (Stansbury et al., 2013), or co-occurrence values from large text corpora (Mitchell et al., 2008; Turney and Pantel, 2010; Wehbe et al., 2014; Huth et al., 2016). This type of model would have several advantages over binary decoders. First, a feature-based decoder could improve generalization because it would only require that all the features were present in the estimation stimulus, and not necessarily that every individual category was present. Second, the HLR model assumes that each category is independent of every other category given its hypernyms. This assumption is clearly false in many cases (Blei et al., 2003; Stansbury et al., 2013). For example, although *car* and *road* are distant relatives in the WordNet taxonomy, they are highly correlated in natural stimuli. A decoder that takes these statistical relationships into account could combine information from categories that are not directly related in the WordNet taxonomy, which would potentially improve decoding performance.

In recent years, the field of brain reading has generated considerable interest from scientists and the public alike. Every improvement in brain measurement technology brings us closer to the goal of a general device for reading out the state of a person's brain. To that end, the HLR model developed here has improved our ability to simultaneously decode many variables while respecting some of the statistical dependencies between them. Yet there are still many issues in brain reading that remain unsolved. We believe that the most important theoretical limitation is that all current methods (the HLR included) assume independence between variables that are actually not independent. One example is the assumption that each category within a scene occurs independently, as discussed above. Another example is the assumption that stimuli are independent from timepoint to timepoint. Relaxing these assumptions should improve the performance of future brain decoders. The future ideal decoder should capture as many of these dependencies among stimulus variables as possible, thus minimizing the amount of information needed to decode the stimuli.

## Author contributions

AH and TL designed and carried out the analysis, with input from NB, JG, SN, and AV. AH, NB, AV, and SN collected the data. SN designed the stimulus. AH labeled semantic categories in the stimulus. AH and TL wrote the paper, with contributions from NB, SN, JG, and AV. JG oversaw all stages of research.

### Conflict of interest statement

The authors declare that the research was conducted in the absence of any commercial or financial relationships that could be construed as a potential conflict of interest.
