# Supplementary material for: Decoding the Semantic Content of Natural Movies from Human Brain Activity
Source: Front Syst Neurosci. 2016 Oct 7;10:81. doi: 10.3389/fnsys.2016.00081 (PMC5057448; doi:10.3389/fnsys.2016.00081)
Supplement: Supplementary file 2 [file Presentation1.PDF]

Supplementary Information for
Decoding the semantic content of natural movies from human brain activity

Alexander G. Huth, Tyler Lee, Shinji Nishimoto, Natalia Y. Bilenko, An T. Vu, Jack L. Gallant

---

Table of Contents

Supplementary Tables
1. Top 30 significantly decoded categories across all subjects
2. WordNet connections that are significantly reflected in brain activity or significantly not reflected in brain activity

Supplementary Figures
1. Decoding results for subject AH
2. Decoding results for subject AV
3. Decoding results for subject JG
4. Decoding results for subject ML
5. Decoding results for subject NB
6. Decoding results for subject TC
7. Decoding results for subject WH
8. Overall decoding performance of each time point versus scene complexity

Supplementary Movies
1. Movie of decoded categories and stimuli

Supplementary Table 1. Top 30 significantly decoded categories across all subjects

**Category**	**AUC**	**p-value**
Skid.v.04	0.97	2.22E-016
Table.n.02	0.97	2.22E-016
Run.v.01	0.96	2.22E-016
Lion.n.01	0.96	2.22E-016
Big_cat.n.01	0.96	2.22E-016
Black.n.01	0.95	2.22E-016
Achromatic_color.n.01	0.95	2.22E-016
Color.n.01	0.95	2.22E-016
Visual_property.n.01	0.95	2.22E-016
Property.n.02	0.95	2.22E-016
Mechanism.n.05	0.95	6.12E-009
Bounce.v.01	0.95	3.49E-009
Slope.n.01	0.93	2.22E-016
Helicopter.n.01	0.93	7.50E-007
Sun.n.01	0.93	2.22E-016
Beach.n.01	0.93	2.42E-013
Hotel.n.01	0.92	2.22E-016
Talk.v.02	0.92	2.22E-016
Guard.n.01	0.92	1.66E-010
Watchman.n.01	0.91	3.73E-010
Water.n.01	0.91	2.22E-016
Seaweed.n.01	0.91	3.11E-006
Defender.n.01	0.91	4.24E-009
Act.v.01	0.91	2.22E-016
Communicate.v.02	0.91	2.22E-016
Liquid.n.03	0.91	2.22E-016
Microorganism.n.01	0.9	2.97E-006
Fire.n.01	0.9	2.22E-016
Whole.n.02	0.9	2.22E-016
Mercantile_establishment.n.01	0.9	3.20E-011

Supplementary Table 2. WordNet category groupings that are not reflected in brain activity

Edge	p-value
thing.n.12 → body_part.n.01	3.11E-012
lake.n.01 → pond.n.01	2.22E-004
organism.n.01 → plant.n.02	6.04E-007
abstraction.n.06 → communication.n.02	2.82E-005
material.n.01 → dust.n.01	4.15E-009
object.n.01 → land.n.04	1.35E-005
shape.n.02 → round_shape.n.01	4.44E-006
equine.n.01 → horse.n.01	4.50E-006
seabird.n.01 → penguin.n.01	3.64E-004
physical_entity.n.01 → process.n.06	8.54E-006
gesticulate.v.01 → nod.v.01	3.14E-008
fluid.n.02 → gas.n.02	1.22E-009
way.n.06 → road.n.01	4.00E-004
activity.n.01 → application.n.03	1.39E-006
move.v.02 → pour.v.01	3.77E-007
whole.n.02 → natural_object.n.01	0.00E+000
group.n.01 → biome.n.01	1.06E-004

Supplementary Figures

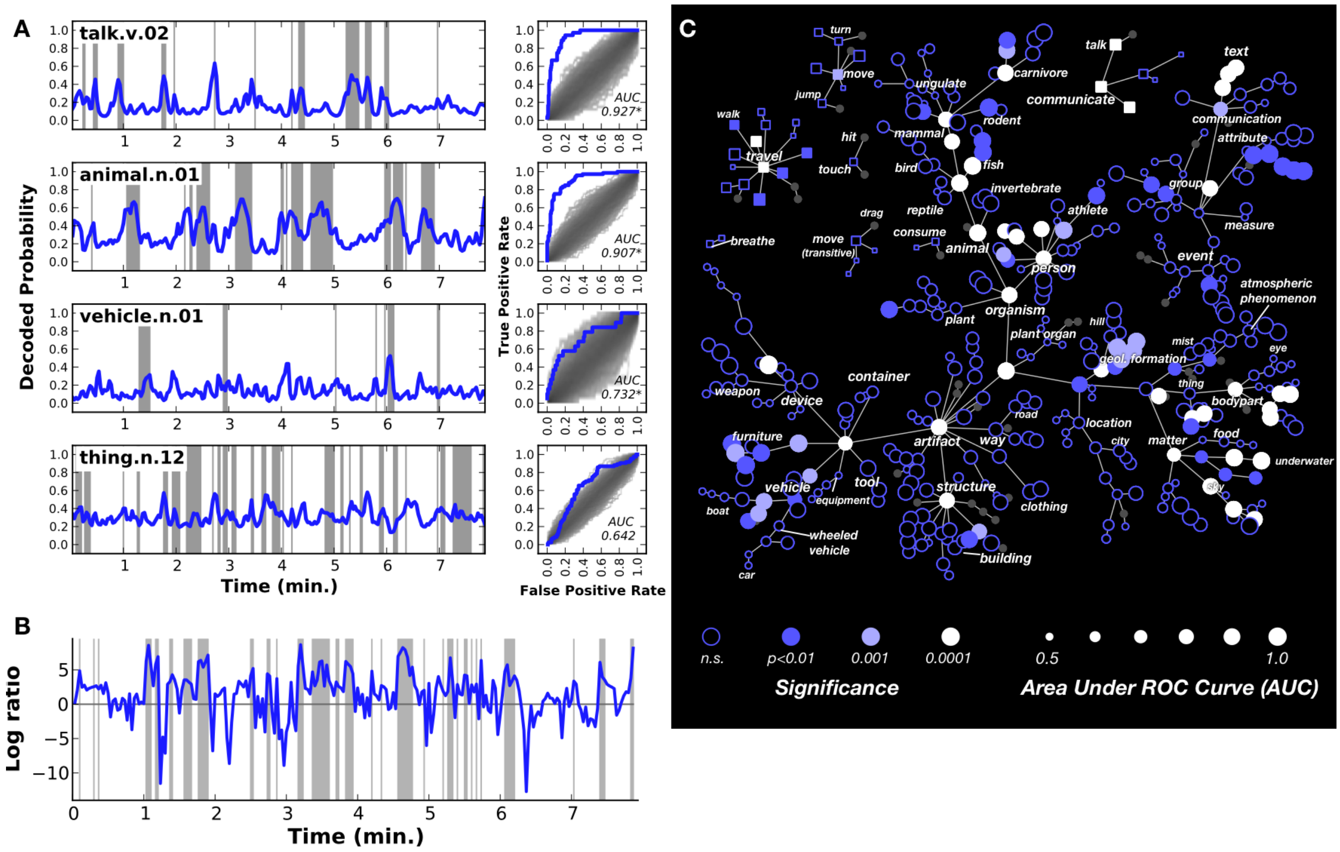

Supplementary Figure 1. Decoding results for subject AH. (**A**) Similar to Figure 3 in the main text, this figure shows decoding results for four out of the 479 categories decoded in this study using the data from subject AH. The left column shows the decoded probability that each category is present (blue line) and times when the category is actually present (shaded regions). The right column shows ROC analyses for these four categories. For details see Figure 3. (**B**) Similar to Figure 7 in the main text, this figure shows how well all categories are decoded at each time point for subject AH. Decoding accuracy is expressed as the log likelihood of the actual category labels given the decoding model, relative to the prior likelihood that each category is present. Values greater than zero indicate that the labels are more likely under the decoding model than the prior model. Shaded regions indicate performance significantly better than chance (p<0.01 uncorrected, permutation test). For details see Figure 7. (**C**) Similar to Figure 4 in the main text, this figure shows the decoding accuracy (quantified using AUC) for each of the 479 categories decoded in this study in subject AH, arranged according to the graphical structure of WordNet. The size of each marker corresponds to the AUC of the decoder for that category, and the color shows the *p*-value of the decoder. For details see Figure 4.

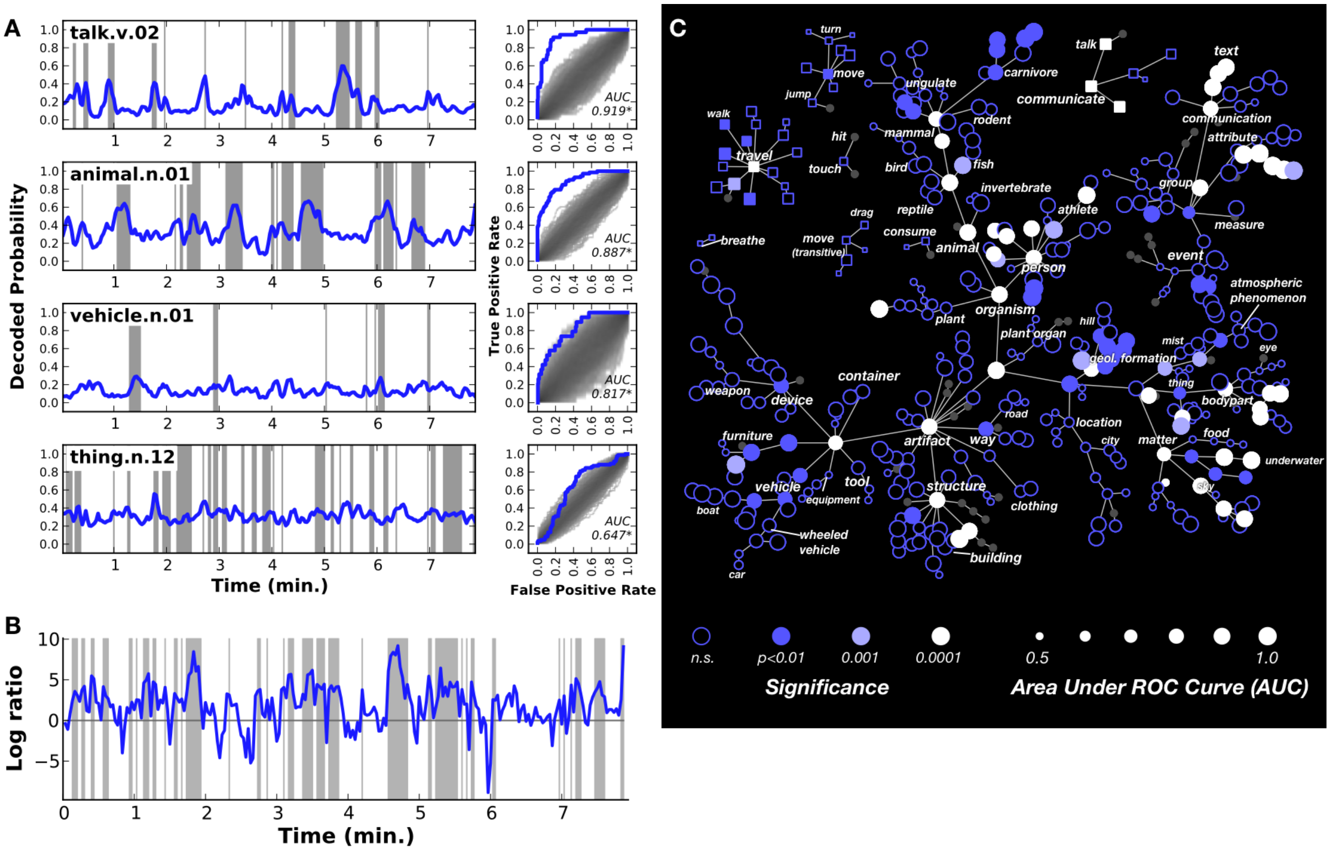

Supplementary Figure 2. Decoding results for subject AV. (**A**) Similar to Figure 3 in the main text, this figure shows decoding results for four out of the 479 categories decoded in this study using the data from subject AV. The left column shows the decoded probability that each category is present (blue line) and times when the category is actually present (shaded regions). The right column shows ROC analyses for these four categories. For details see Figure 3. (**B**) Similar to Figure 7 in the main text, this figure shows how well all categories are decoded at each time point for subject AV. Decoding accuracy is expressed as the log likelihood of the actual category labels given the decoding model, relative to the prior likelihood that each category is present. Values greater than zero indicate that the labels are more likely under the decoding model than the prior model. Shaded regions indicate performance significantly better than chance (p<0.01 uncorrected, permutation test). For details see Figure 7. (**C**) Similar to Figure 4 in the main text, this figure shows the decoding accuracy (quantified using AUC) for each of the 479 categories decoded in this study in subject AV, arranged according to the graphical structure of WordNet. The size of each marker corresponds to the AUC of the decoder for that category, and the color shows the *p*-value of the decoder. For details see Figure 4.

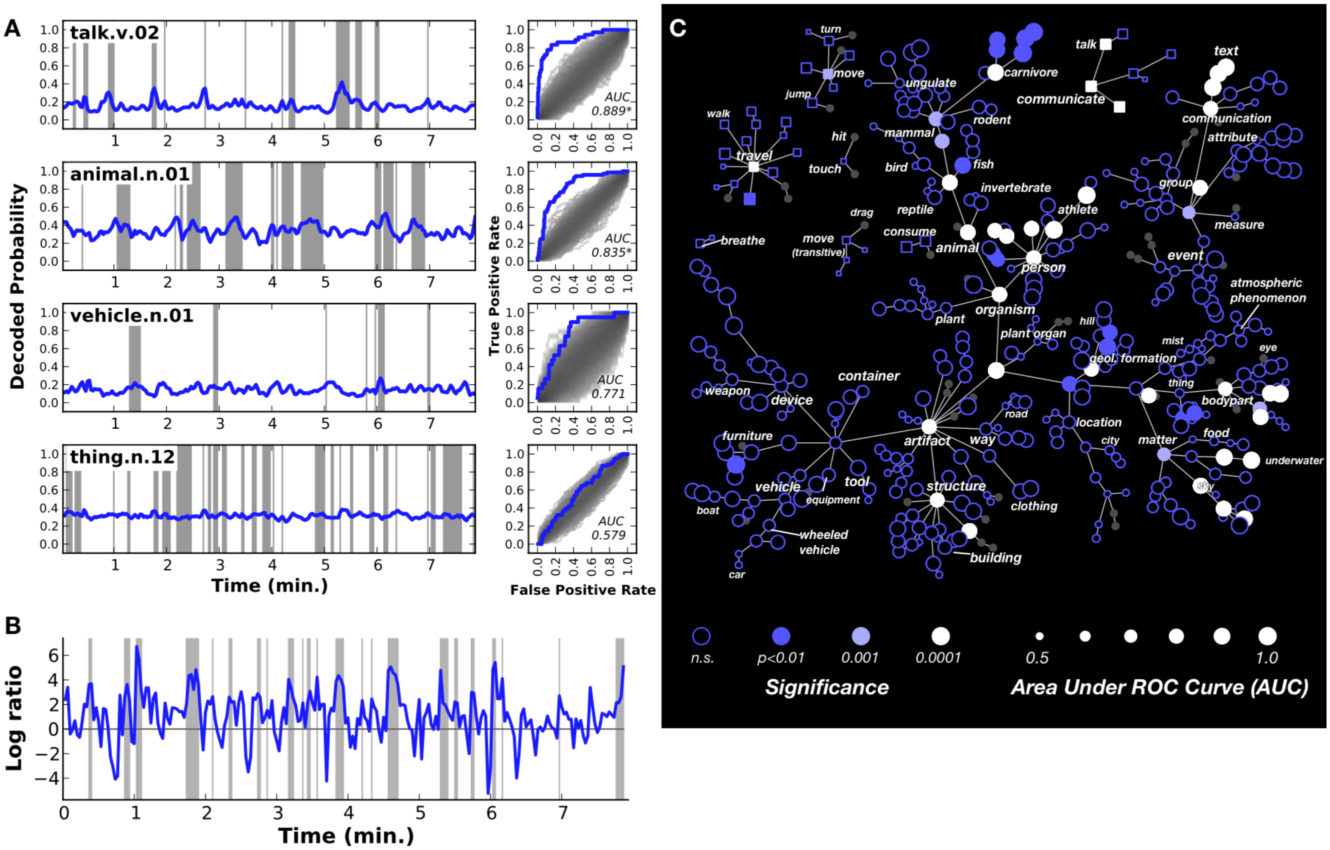

Supplementary Figure 3. Decoding results for subject JG. (**A**) Similar to Figure 3 in the main text, this figure shows decoding results for four out of the 479 categories decoded in this study using the data from subject JG. The left column shows the decoded probability that each category is present (blue line) and times when the category is actually present (shaded regions). The right column shows ROC analyses for these four categories. For details see Figure 3. (**B**) Similar to Figure 7 in the main text, this figure shows how well all categories are decoded at each time point for subject JG. Decoding accuracy is expressed as the log likelihood of the actual category labels given the decoding model, relative to the prior likelihood that each category is present. Values greater than zero indicate that the labels are more likely under the decoding model than the prior model. Shaded regions indicate performance significantly better than chance ($p<0.01$ uncorrected, permutation test). For details see Figure 7. (**C**) Similar to Figure 4 in the main text, this figure shows the decoding accuracy (quantified using AUC) for each of the 479 categories decoded in this study in subject JG, arranged according to the graphical structure of WordNet. The size of each marker corresponds to the AUC of the decoder for that category, and the color shows the *p*-value of the decoder. For details see Figure 4.

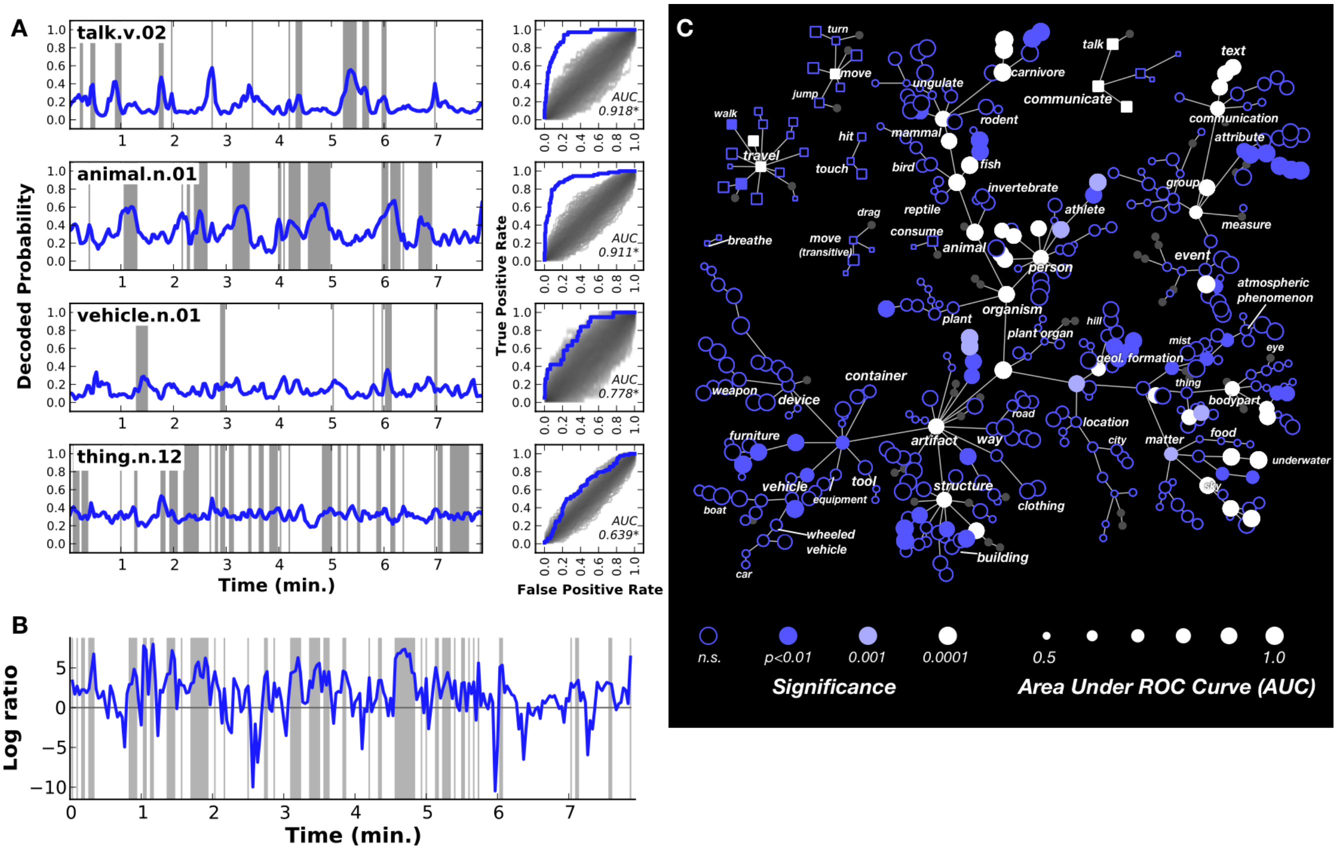

Supplementary Figure 4. Decoding results for subject ML. (**A**) Identical to Figure 3 in the main text, this figure shows decoding results for four out of the 479 categories decoded in this study using the data from subject ML. The left column shows the decoded probability that each category is present (blue line) and times when the category is actually present (shaded regions). The right column shows ROC analyses for these four categories. For details see Figure 3. (**B**) Similar to Figure 7 in the main text, this figure shows how well all categories are decoded at each time point for subject ML. Decoding accuracy is expressed as the log likelihood of the actual category labels given the decoding model, relative to the prior likelihood that each category is present. Values greater than zero indicate that the labels are more likely under the decoding model than the prior model. Shaded regions indicate performance significantly better than chance (p<0.01 uncorrected, permutation test). For details see Figure 7. (**C**) Similar to Figure 4 in the main text, this figure shows the decoding accuracy (quantified using AUC) for each of the 479 categories decoded in this study in subject ML, arranged according to the graphical structure of WordNet. The size of each marker corresponds to the AUC of the decoder for that category, and the color shows the *p*-value of the decoder. For details see Figure 4.

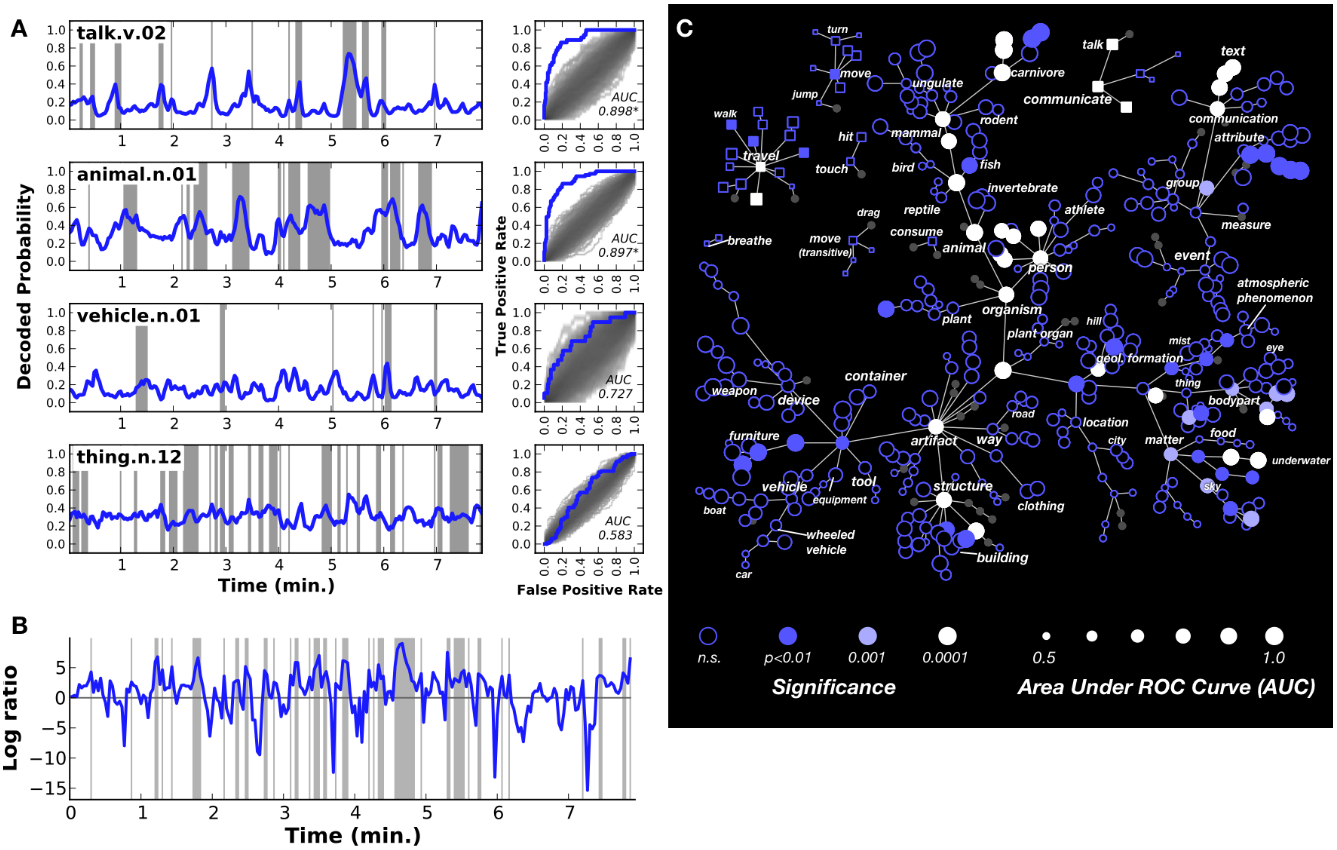

Supplementary Figure 5. Decoding results for subject NB. (**A**) Similar to Figure 3 in the main text, this figure shows decoding results for four out of the 479 categories decoded in this study using the data from subject NB. The left column shows the decoded probability that each category is present (blue line) and times when the category is actually present (shaded regions). The right column shows ROC analyses for these four categories. For details see Figure 3. (**B**) Similar to Figure 7 in the main text, this figure shows how well all categories are decoded at each time point for subject NB. Decoding accuracy is expressed as the log likelihood of the actual category labels given the decoding model, relative to the prior likelihood that each category is present. Values greater than zero indicate that the labels are more likely under the decoding model than the prior model. Shaded regions indicate performance significantly better than chance (p<0.01 uncorrected, permutation test). For details see Figure 7. (**C**) Similar to Figure 4 in the main text, this figure shows the decoding accuracy (quantified using AUC) for each of the 479 categories decoded in this study in subject NB, arranged according to the graphical structure of WordNet. The size of each marker corresponds to the AUC of the decoder for that category, and the color shows the *p*-value of the decoder. For details see Figure 4.

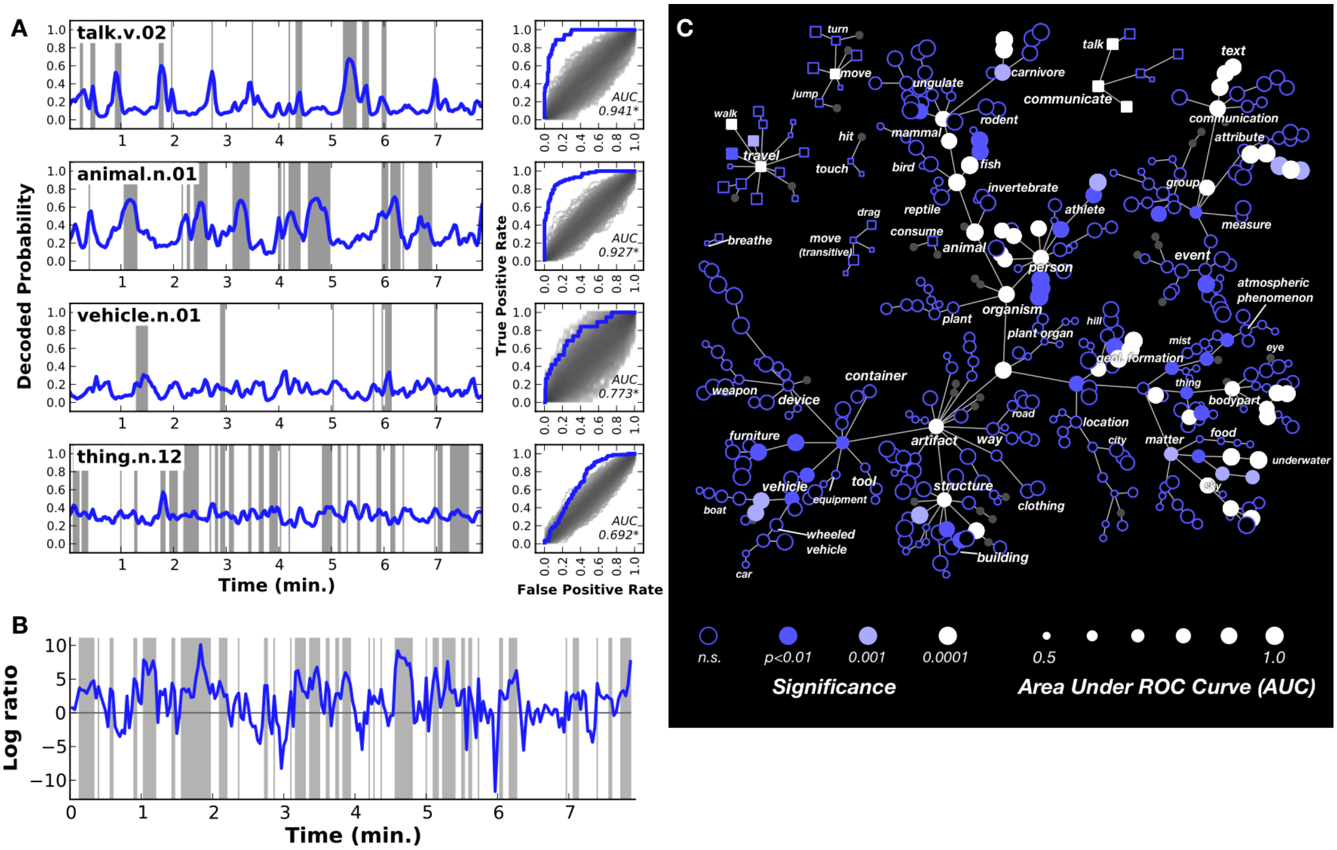

Supplementary Figure 6. Decoding results for subject TC. (**A**) Similar to Figure 3 in the main text, this figure shows decoding results for four out of the 479 categories decoded in this study using the data from subject TC. The left column shows the decoded probability that each category is present (blue line) and times when the category is actually present (shaded regions). The right column shows ROC analyses for these four categories. For details see Figure 3. (**B**) Similar to Figure 7 in the main text, this figure shows how well all categories are decoded at each time point for subject TC. Decoding accuracy is expressed as the log likelihood of the actual category labels given the decoding model, relative to the prior likelihood that each category is present. Values greater than zero indicate that the labels are more likely under the decoding model than the prior model. Shaded regions indicate performance significantly better than chance (p<0.01 uncorrected, permutation test). For details see Figure 7. (**C**) Similar to Figure 4 in the main text, this figure shows the decoding accuracy (quantified using AUC) for each of the 479 categories decoded in this study in subject TC, arranged according to the graphical structure of WordNet. The size of each marker corresponds to the AUC of the decoder for that category, and the color shows the *p*-value of the decoder. For details see Figure 4.

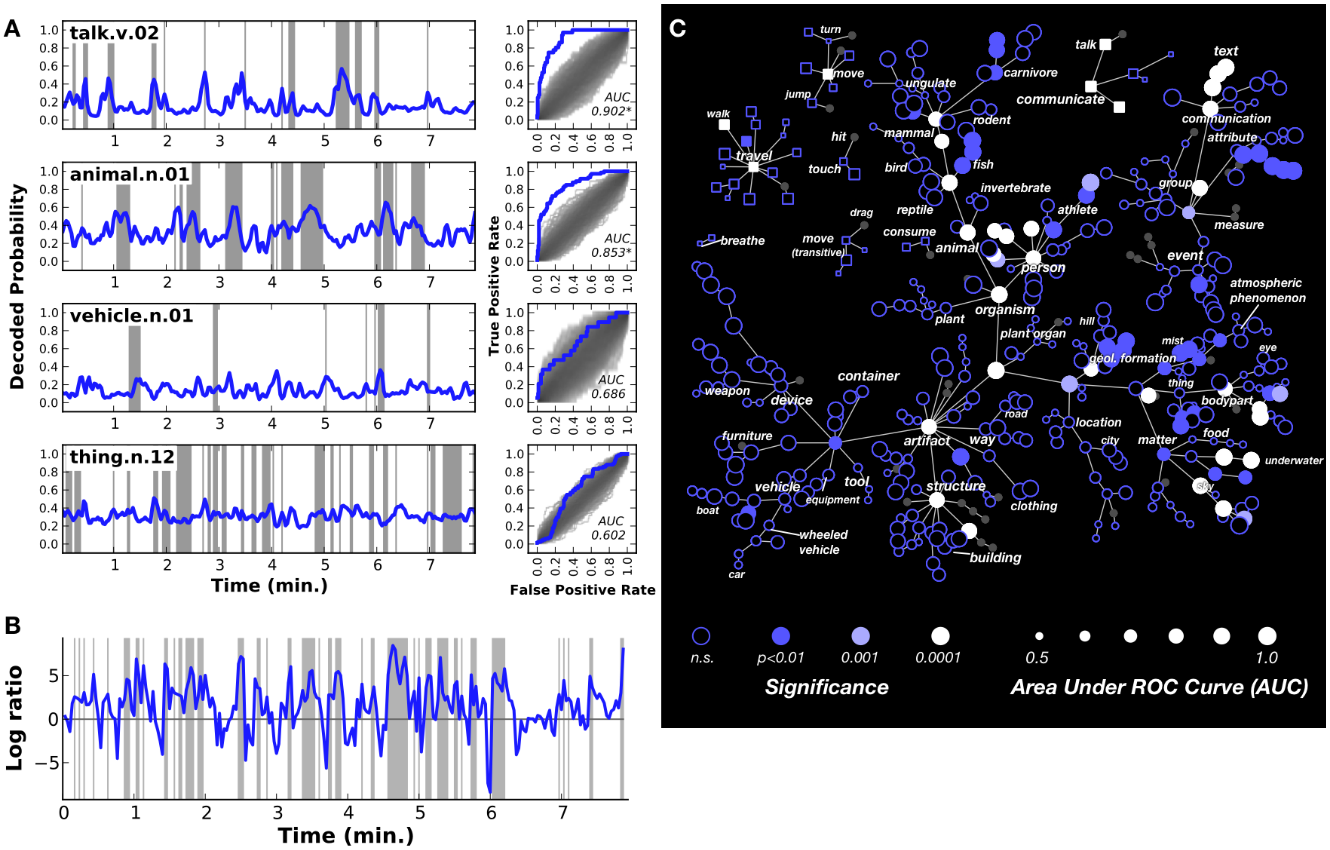

Supplementary Figure 7. Decoding results for subject WH. (**A**) Similar to Figure 3 in the main text, this figure shows decoding results for four out of the 479 categories decoded in this study using the data from subject WH. The left column shows the decoded probability that each category is present (blue line) and times when the category is actually present (shaded regions). The right column shows ROC analyses for these four categories. For details see Figure 3. (**B**) Similar to Figure 7 in the main text, this figure shows how well all categories are decoded at each time point for subject WH. Decoding accuracy is expressed as the log likelihood of the actual category labels given the decoding model, relative to the prior likelihood that each category is present. Values greater than zero indicate that the labels are more likely under the decoding model than the prior model. Shaded regions indicate performance significantly better than chance ($p<0.01$ uncorrected, permutation test). For details see Figure 7. (**C**) Similar to Figure 4 in the main text, this figure shows the decoding accuracy (quantified using AUC) for each of the 479 categories decoded in this study in subject WH, arranged according to the graphical structure of WordNet. The size of each marker corresponds to the AUC of the decoder for that category, and the color shows the *p*-value of the decoder. For details see Figure 4.

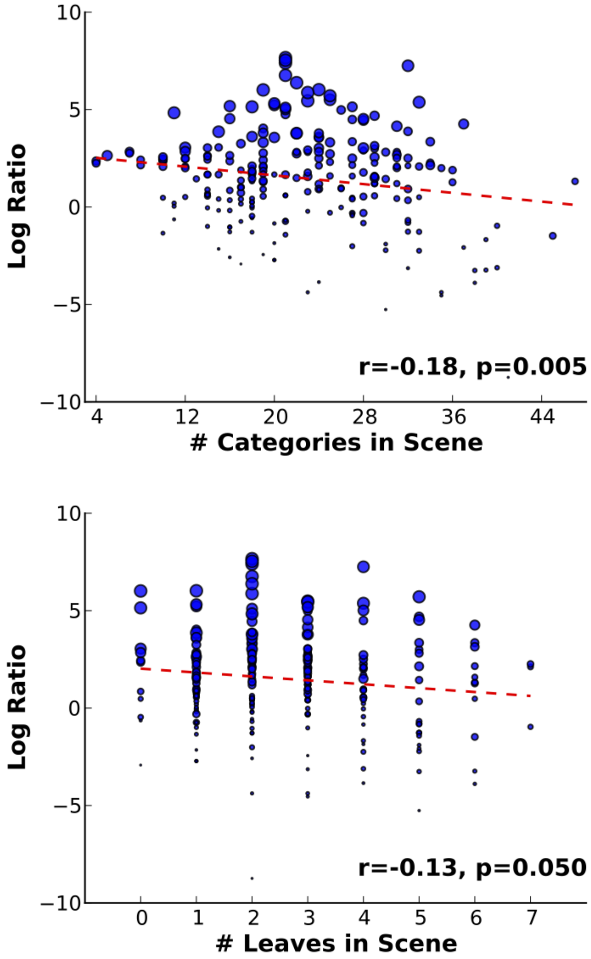

Supplementary Figure 8. Overall decoding performance of each time point versus scene complexity. Decoding accuracy is expressed as the log likelihood of model predictions relative to the prior log likelihood that each category is present. Here we plotted the decoding accuracy as a function of the total number of categories in a scene (**top**) or the number of "leaf nodes" (i.e. excluding hypernyms) in the scene (**bottom**). Marker size shows significance of the likelihood ratio for each scene (marker area is proportional to the negative log of the *p*-value). We found a small but significant negative correlation between decoding accuracy and both measures of scene complexity. This suggests that the HLR decoding model is less successful at decoding complex scenes that contain many categories, and is more successful at decoding simpler scenes. This could reflect nonlinear interactions between different categories, which are not captured by the linear HLR model.
